# Development, feasibility and performance of a health risk appraisal questionnaire for older persons

**DOI:** 10.1186/1471-2288-7-1

**Published:** 2007-01-11

**Authors:** Andreas E Stuck, Kalpa Kharicha, Ulrike Dapp, Jennifer Anders, Wolfgang von Renteln-Kruse, Hans Peter Meier-Baumgartner, Danielle Harari, Cameron G Swift, Katja Ivanova, Matthias Egger, Gerhard Gillmann, Jerilyn Higa, John C Beck, Steve Iliffe

**Affiliations:** 1 Department of Geriatrics, University of Bern, Spital Bern-Ziegler, Morillonstrasse 75-91, CH-3001 Bern, Switzerland; 2 Division of Geriatrics, Department of General Internal Medicine, Insel University of Bern Hospital, CH-3010 Bern, Switzerland; 3 Department of Primary Care and Population Sciences, University College London, Hampstead Campus, Rowland Hill Street, London NW3 2PF, UK; 4 Albertinen-Haus Geriatrics Centre, University of Hamburg, Sellhopsweg 18-22, D-22459 Hamburg, Germany; 5 Department of Ageing and Health, Guys and St Thomas' NHS Foundation Trust, 9th Floor North Wing, St Thomas' Hospital, Lambeth Palace Road, London SE1 7EH, UK; 6 Department of Health Care of the Elderly, Kings College London, Clinical Age Research Unit, King's College Hospital, Bessemer Road, London SE5 9PJ, UK; 7 Department of Social and Preventive Medicine, University of Bern, Finkenhubelweg 11, CH-3012 Bern, Switzerland; 8 Department of Emergency Medicine, University of California, Los Angeles, California, USA; 9 School of Medicine, University of California, Los Angeles, 10833 Le Conte Ave. 32-144, Los Angeles CA-90024-1687, USA

## Abstract

**Background:**

Health risk appraisal is a promising method for health promotion and prevention in older persons. The Health Risk Appraisal for the Elderly (HRA-E) developed in the U.S. has unique features but has not been tested outside the United States.

**Methods:**

Based on the original HRA-E, we developed a scientifically updated and regionally adapted multilingual Health Risk Appraisal for Older Persons (HRA-O) instrument consisting of a self-administered questionnaire and software-generated feed-back reports. We evaluated the practicability and performance of the questionnaire in non-disabled community-dwelling older persons in London (U.K.) (N = 1090), Hamburg (Germany) (N = 804), and Solothurn (Switzerland) (N = 748) in a sub-sample of an international randomised controlled study.

**Results:**

Over eighty percent of invited older persons returned the self-administered HRA-O questionnaire. Fair or poor self-perceived health status and older age were correlated with higher rates of non-return of the questionnaire. Older participants and those with lower educational levels reported more difficulty in completing the HRA-O questionnaire as compared to younger and higher educated persons. However, even among older participants and those with low educational level, more than 80% rated the questionnaire as easy to complete. Prevalence rates of risks for functional decline or problems were between 2% and 91% for the 19 HRA-O domains. Participants' intention to change health behaviour suggested that for some risk factors participants were in a pre-contemplation phase, having no short- or medium-term plans for change. Many participants perceived their health behaviour or preventative care uptake as optimal, despite indications of deficits according to the HRA-O based evaluation.

**Conclusion:**

The HRA-O questionnaire was highly accepted by a broad range of community-dwelling non-disabled persons. It identified a high number of risks and problems, and provided information on participants' intention to change health behaviour.

## Background

There is a growing interest in health risk appraisal (HRA) for use in older persons. HRA instruments typically consist of a questionnaire and an algorithm for generating feedback reports to participants and health care providers. Controlled studies support that HRA combined with supplemental counselling by a physician, health educator, or other health professional is a potentially cost-effective method of health promotion and prevention for older persons [1]. Providers, organisations or researchers interested in a HRA can now choose among multiple HRA instruments that have been developed for use in older persons [1].

**Figure 1:**
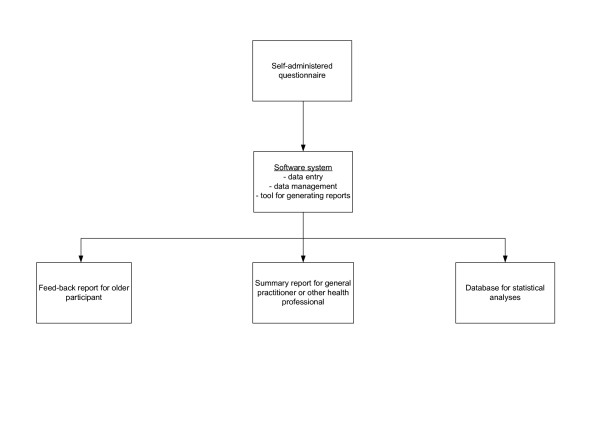
Common components of Health Risk Appraisal for the Elderly (HRA-E) and of all versions of the Health Risk Appraisal for Older Persons (HRA-O)

There are multiple differences between available HRA instruments for use in older persons. The HRA-E (HRA for the Elderly) questionnaire developed by a University of California faculty group has several distinguishing features [1,2]: (1) its main purpose is to identify risk factors for functional decline; this contrasts with other HRA instruments which focus on risk factors for mortality or address selected health behaviour and preventative care issues alone; (2) unlike other HRA instruments, it is based on scientific evidence for the selection of risk factor domains and instruments to measure these domains, and for the definition of the recommendations in the feedback [1]; (3) unlike most other HRA questionnaires for older persons (with the exception of the YOU FIRST Senior Health Assessment [1]), it identifies intention and barriers to changing health behaviours which can be used to enhance tailoring of participant feed-back ; and (4) it includes a computerised algorithm to generate feed-back to both older persons and general practitioners or other health professionals (see Figure 1), in contrast with conventional HRA instruments which do not specifically address primary care practitioners.

However, despite these advantages, there are factors limiting the use of the HRA-E in Europe: first it had not been developed for use in a multilingual environment, second its feasibility and performance had not been tested outside the North American environment, and third, the HRA-E is outdated because new scientific evidence has accumulated since its development in 1997.

**Figure 2:**
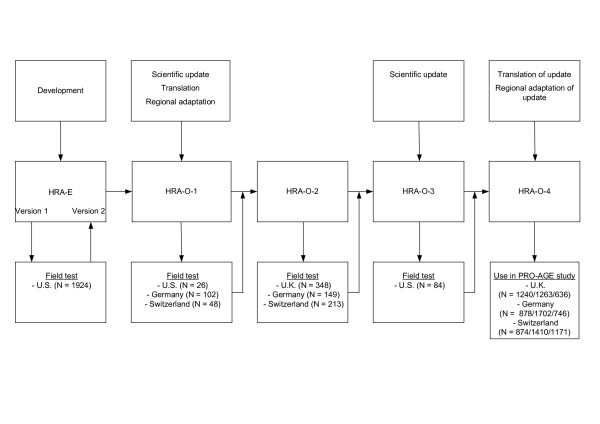
Development stages from the Health Risk Appraisal for the Elderly (HRA-E) to the Health Risk Appraisal for Older Persons version 4 (HRA-O)

We decided to revise and update the original HRA-E and to name this new version "Health Risk Appraisal for Older Persons" (HRA-O) [3]. The stepwise development from HRA-E to HRA-O instrument versions 1 to 4 is described in the following paragraphs, and depicted in Figure 2.

### Development and testing of HRA-E

The original HRA-E was used as a basis for the subsequent development of HRA-O versions. After a multi-step development process including a systematic literature review, expert input, as well as multiple focus group and pilot testing activities, a first version of the HRA-E was developed. It consisted of a self-administered questionnaire (for the 17 included domains, see Table 1), a software program for generating an individualised feed-back report to the older participant and a summary report for the health care professional [2]. This first version was tested in three samples of older Americans: (1) a large medical group practice with links to Health Maintenance Organization (HMO) plans, (2) a national sample of American Association of Retired Persons (AARP) members, and (3) a senior centre (total N = 1924) [2]. A second version of the HRA-E was generated based on this experience.

**Table 1:** Sources of Health Risk Appraisal for the Elderly (HRA-E) and Health Risk Appraisal for Older Persons (HRA-O) questionnaire

**Domain**	**Definition of problem risk**	**Description of questions (questionnaire item source)**
Accident prevention	Driving without using seat belt	Use of seatbelt [26]
Alcohol use	Possible hazardous alcohol use (based on age- and gender-specific limits of quantity and frequency of self-reported alcohol use)	The WHO Alcohol Use Disorders Identification Test [15]
Falls	History of repeated falls in previous 12 months	Study of Osteoporotic Fractures Research Group Survey [20]
	Self-reported limitation of activities due to fear of falling	Fear of falling [32]
Functional status	Difficulty/need for human assistance in ≥1 BADL item	Basic activities of daily living (BADL) [19]
	Difficulty/need for human assistance in ≥2 IADL items	Instrumental activities of daily living (IADL) [22]
	Changed kind of activity	Preclinical mobility disability [13]*
	Decreased frequency of activity	
Health status	Moderate or fair self-perceived health status	Self-perceived health status [18]
Hearing	Impaired hearing	Hearing Handicap Inventory for the Elderly [23]
	No hearing check-up in previous year	History of hearing examination [33]
Incontinence	Urinary incontinence on > 5 days during the last year	Urinary incontinence (Medical, Epidemiological and Social Aspects of Aging Project Questionnaire) [17]
Medication use	Use of ≥4 medications	Use of medications [2]
	Use of long-acting benzodiazepine or amitriptyline	Inappropriate medication use [14] *
	Medication side effect	Medication side effects [35]
	Difficulties with medication compliance	Medication compliance [2]
Medical History	Presence of chronic condition(s)	Chronic conditions [18]
Memory	Memory problems	Memory Self Report [28]
Mood	Depressive mood	5-item Mental Health Inventory Screening Test [29]
Nutrition	Consumption of >2 high fat food items per day	Cholesterol Reduction in Seniors Program Fat Food Screening
		Questionnaire [31]
	Consumption of < 5 fruit/fibre items per day	Cholesterol Reduction in Seniors Program Plant Food Screening
		Questionnaire [31]*
	Motivation for change in fat intake/fruit intake	Transtheoretical model of behaviour change [2, 21,27]
	Body mass index <20 or ≥27; loss of weight	Self-reported height and weight (body mass index), weight change
Oral Health*	Oral health problem	Geriatric oral health assessment index [11]*
	No dental check in previous year	History of dental care [33]
Pain*	Presence of moderate to severe pain	Geriatric Pain Measure [12]*
Physical activity	Less than 5 times/week moderate or strenuous activity	Physical Activity Scale for the Elderly [34]
	Motivation for behaviour change	Transtheoretical model of behaviour change [2,21,27]
Preventative care	No blood pressure control in previous year	History of blood pressure measurement [33]
	Elevated self-reported blood pressure	Self-reported blood pressure
	No mammography in previous 2 years	History of breast cancer screening [33]
	No cervical smear in previous 3 years	History of cervical smear [33]
	No cholesterol measurement in previous 5 years	History of cholesterol measurement [33]
	Elevated self-reported cholesterol	Self-reported cholesterol level
	No faecal occult blood test in previous year	History of colon cancer screening [33]
	No blood glucose measurement in previous 3 years	History of diabetes screening [33]
	No influenza vaccination in previous year	History of influenza immunisation [33]
	No pneumococcal vaccination (ever)	History of pneumococcal immunisation [33]
Social factors	Low level of emotional support	Medical Outcomes Study Social Support Survey [29]
	High risk of social isolation	Lubben Social Network Scale [24]
	Marginal family ties	Subscale Lubben Social Network Scale [24]
	Marginal friendship ties	Lubben Social Network Scale [24]
	No participation in groups	Single-item question [16]
Tobacco use	Current tobacco use	Tobacco use (Partners in Prevention Tobacco Use Questionnaire) [2]
	Motivation for behaviour change	Transtheoretical model of behaviour change [2,21,27]
Vision	Problem in ≥ 1 vision sub-domain	Visual Functioning Questionnaire [25]*
	No vision check-up in previous year	History of vision examination [33]

In addition, the questionnaire includes socio-demographics survey items (age, gender, education, professional activity, living arrangement) and a survey for participant feed-back to the questionnaire. Items marked with an asterisk (*) were added to the HRA-O questionnaires and are NOT included in the HRA-E.

### Scientific update of HRA-E

The first step involved a scientific update of the HRA-E. First, based on a systematic literature search on risk factors for functional status decline in older persons [4], potential new domains for inclusion in the updated questionnaire were identified. This review was presented to an international Expert Panel (from Denmark, Germany, Netherlands, Switzerland, U.K., USA) in the fields of epidemiology, geriatrics, sociology and nursing. We used a consensus panel process adapted to a multilingual group of experts. They evaluated new potential domains based on the four criteria listed below, which had previously been used for the development of the original HRA-E instrument:

(1) magnitude of effect and potential impact on functional impairment;

(2) validity and generalisability of results;

(3) potential for risk reduction;

(4) feasibility of assessment.

In a second step, the same Expert Panel selected instruments to measure new potential domains, and decided whether existing instruments in the original HRA-E questionnaire had to be replaced. A list of instruments to be considered was developed for each domain of the updated questionnaire, in conjunction with information from the current literature informing the experts about the validity and reliability of each instrument for use in community-dwelling older people. The following criteria for selecting the instruments or evaluating the inclusion of existing instruments were used:

(1) reliability;

(2) validity;

(3) feasibility; and

(4) use of the instrument in other large databases of older persons.

### Development of HRA-O version 1 (HRA-O-1)

Based on recommendations of the Expert Panel, specific questionnaires for two domains (on fruit/fibre intake and vision function, details in Table 1) were added to the new HRA-O-1 questionnaire. In addition, selected improvements were made for individual questionnaire items and recommendations in the feed-back statements.

In a next step, the questionnaire and text for the participant and provider feed-back reports were translated into the German language by a professional translator, and then translated back to the English language by a second translator who was blinded to the original version. The back-translation was compared with the original version, and discrepancies were resolved by a third independent translator. Based on this version intended for use in Germany, we developed a Switzerland German version by adapting language and grammar. Also, based on the American English version, a separate English version for use in the U.K. was developed, following changes to the language, grammar and style. These translations required a redesign of the original HRA-E to accommodate multiple language versions. This involved a revision of the questionnaire data entry system and of the software system generating the feed-back reports. The newly generated HRA-O-version-1 (HRA-O-1) was alpha tested by evaluating the functionality and content of data entry and report generation.

Based on this HRA-O-1, focus group meetings with older persons and general practitioners, and pilot tests in small groups of older persons in Switzerland, Germany, and the U.K., were conducted. The questionnaire was then regionally adapted without changing the content of the questions. For example, units for reporting weight (e.g., stones, kilograms) or examples of food items with a high fat content (e.g. hot dogs in U.K., and "Bratwurst" in Switzerland) were added as needed. The feed-back statements for the four language versions (American English, U.K. English, Germany German, and Switzerland German) were adapted to incorporate regional variations including postal addresses, referrals to health providers, and access or payment for preventative care services.

### Testing of HRA-O-1

A field test in 26 community-dwelling older persons in the US evaluated the functionality and acceptance of HRA-O-1. In addition, we also conducted a study for evaluating the reliability of instruments included in the HRA-O-1 questionnaire (Table 1) in three samples of community-dwelling persons aged 75 years and older in Hamburg (Germany) (N = 51), Ulm (Germany) (N = 51) and Bern (Switzerland) (N = 48) [5]. In a first sub-sample of 100 persons, the test-retest reliability of individual questionnaires included in HRA-O-1 (Table 1) and of specific questionnaires on oral health and pain (two domains that we considered adding to the HRA-O-1 based on the Expert Panel recommendations) [6]. Test-retest reliability was good to excellent, as measured by Cohen's Kappa (0.64 ≤ κ ≤ 0.89) [7], with the following exceptions. For three domains (pain, preventative care, and falls), Cohen's Kappa was <0.6. In a different sub-sample of 50 persons, the validity of the specific questionnaires included in the HRA-O-1 questionnaire was determined by comparing self-administered with interviewer-administered answers to the questionnaires [7]. Cohen's Kappa revealed good to excellent validity in most domains with values ranging between 0.69 and 1.0. Values were below 0.69 for questionnaires assessing physical activity, oral health, and basic activities of daily living. Low Kappa values could be explained by suboptimal presentation of the questions in the self-administered questionnaire, and consequently, introductory statements, wording of these questions and graphical presentation of items were improved.

### Testing of HRA-O-version-2 (HRA-O-2)

Based on the testing of HRA-O-1, a revised version HRA-O-2 was developed. The feasibility of this updated version was then tested in three selected samples of persons aged 65 years and older in three European countries (U.K.: urban-based general practitioner lists in London, N = 348; Germany: occupants of sheltered housing facilities in Hamburg, N = 149; Switzerland: community-based lists in rural/suburban area in the Cantons of Solothurn and Bern, N = 213) [8]. The majority of people judged the questionnaire as easy to comprehend (U.K., 81.4%; Germany, 93.1%; Switzerland, 97.2%) and to complete (83.2%, 91.4%, and 95.8%, respectively). Feed-back from older persons to the participant reports was systematically evaluated [9].

We decided to further test the validity of self-reported information on preventative care use captured by HRA-O-2. For this purpose, in the Swiss sub-sample (N = 213), self-reported data of preventative care were compared with medical record based information obtained from general practitioners. Agreement between the two data sources was good to excellent with agreement rates eighty percent or more for the comparison between self-reported and record-based information for the individual measures of preventative care [10].

### Development of HRA-O-version-3 (HRA-O-3)

Based on the original recommendations of the Expert Panel, two new domains were added to the revised HRA-O-3 and measured using the following tools: (1) oral health: the Geriatric Oral Health Assessment Index (GOHAI) was added to assess the impact of oral conditions on physical and psychosocial functions [11] and (2) pain: the multidimensional Geriatric Pain Measure was selected to evaluate the experience and intensity of pain, the impact of pain on function and on social network [12]. Instruments were updated for two domains already included in previous HRA-O versions: (1) inappropriate medication use: we added questions on inappropriate medications, and selected from drugs with potentially severe side effects, the two categories that had the highest prevalence of use in a community-based sample of older persons [13]; (2) preclinical functional decline: a measure of preclinical functional decline was added to identify people early in the trajectory of functional status decline [14]. In addition, information contained in the feed-back reports was updated.

### Testing of HRA-O-3

In order to test the functionality and feasibility of the updated HRA-O-3, a field test of the American English version was conducted in conjunction with the Center for Healthy Aging in Santa Monica, CA, U.S.A. Overall, 84 community-dwelling older persons completed the new HRA-O-3 questionnaires, received feed-back reports, and were asked to provide feed-back on the HRA-O-3. Results confirmed functionality and acceptability, and only minimal remaining areas of improvement were found at this stage. Furthermore persons who recalled the earlier field tests with the original HRA-E instrument confirmed that updates had resulted in improvement related to ease of administration and completeness of contents.

### Development and description of HRA-O-version-4 (HRA-O-4)

Translations and back-translations of newly added components, as well as regional adaptations were made. Based on the testing of HRA-O-3 in the U.S. setting, necessary revisions and adaptations were implemented. As a result, HRA-O-4 was produced in an American English, U.K. English, Germany German, and Switzerland German version (for components, see Figure 1). The U.K. English version of this version 4 of HRA-O questionnaire is enclosed [see Additional file 1]. The 19 domains included in the HRA-O-4 questionnaire are listed in Table 1[11-35].

The generation of feed-back reports to older participants and health care providers is based on a computerised system, including a screen-guided system for manual data entry, and an automated analysis of the entered data. From the entered data, a software system generates summary or risk scores and corresponding detailed written feed-back on identified risks to the older person. The report summarises the suspected problem (while always emphasising that this information is based on self-report) and generates feedback by selecting and arranging words and sentences from more than 1000 possible feedback algorithms. Feed-back reports were developed using current scientific evidence related to health promotion, risk factor modification, and problem management. The summary feed-back report to the health care provider is arranged in the format of a check-list on one double sided page. The older person's report (approximately 32 pages) is personalised and contains both general information on each domain as well as individualised specific recommendations derived from the questionnaire analysis. Cross-links were made between domains, for example by taking into account level of physical activity and body mass index when giving recommendations to an older person reporting high blood pressure management. Participants' intention to change health behaviour or self reported reasons for not changing behaviour was taken into account. In addition, feed-back reports to older participants also included sources of additional information.

### Goals of this study

The goal of this paper is to evaluate the feasibility and performance of the newly developed HRA-O questionnaire with base-line data from an international randomised study, the PRO-AGE study (PRevention in Older people – Assessment in GEneralists' practices) [3]. To evaluate its feasibility, we analysed response rates and feed-back from older persons, including subgroups of older persons in whom use of a self-administered questionnaire is often queried: those with a low level of education and the very old. To evaluate the performance of the HRA-O questionnaire, we determined prevalence rates of identified risks and problems and participants' intention to change health behaviour as well as self-perceived barriers to change.

## Methods

### Study participants

Data presented in this paper are from a randomised controlled study, the PRO-AGE study (London, U.K.; Hamburg, Germany; and Solothurn, Switzerland). In this study, non-disabled community-dwelling older persons were recruited from primary care and randomised to intervention and control groups [3]. After randomisation, all subjects allocated to the intervention group were sent the HRA-O questionnaire. This study examined the effects of the HRA-O linked with a site-specific reinforcement (i.e. supplemental counselling by a physician, health educator, or other health professional) on self reported health behaviour and use of preventative care. The ethical approval of the PRO-AGE project was from the Brent Medical Ethics Committee and King's College Hospital Research Ethics Committee (London), the Ethics Committee of the Ärztekammer Hamburg (Hamburg) and the Kantonale Ethikkommission Solothurn (EKO 0023) (Solothurn).

### Data collection

Prior to randomisation, all study participants completed a screening Pra-questionnaire (Probability of repeated admissions questionnaire) [36], providing information on selected base-line characteristics of study participants. Based on this questionnaire, a Pra risk score [36] was calculated to define a priori risk strata in the randomised controlled study.

Older persons were posted the HRA-O questionnaire and asked to return the completed questionnaire to their general practitioners. For budgetary reasons, no reminders were sent to older persons who did not return the HRA-O questionnaire. The HRA-O questionnaire contained the items listed in Table 1 plus, at the end, a brief survey on participant feed-back to the questionnaire.

### Statistical analyses

Analyses were conducted according to an a priori analytic plan. Base-line characteristics of persons who returned the HRA-O questionnaire ("responders") were compared with those of non-responding persons ("non-responders") using available pre-randomisation data. P-values for differences in base-line characteristics were derived from multivariable logistic regression analyses with a covariate pool consisting of the individual base-line items. P-values for differences in the Pra score between responders and non-responders were derived from t-tests.

Feed-back to the HRA-O questionnaire was compared between participants with higher and lower educational level, and between participants older and younger than 75 years. Categorical and binary outcome data are analysed using Fisher's exact tests, continuous outcome data are compared using t-tests if normally distributed, Mann-Whitney U test if skewed. Data were analysed using the SAS program [37].

## Results

### Response to HRA-O questionnaire

The numbers (percentage) of persons returning the HRA-O questionnaire were 1090 (87.9%) in London, 804 (91.6%) in Hamburg, and 748 (85.6%) in Solothurn. Table 2 compares the characteristics of responders and non-responders to the HRA-O questionnaire. At all sites, persons with fair or poor self-perceived health status were less likely to return the HRA-O questionnaire compared to persons with good or very good self-perceived health status. In Solothurn, this difference was small and statistically non-significant. In London and Hamburg, this difference was larger and statistically significant. In Hamburg, participant age was also related to HRA-O questionnaire response, with older participants having a lower return rate as compared to younger participants. No other characteristics affecting response were identified among the three sites. Overall Pra risk status was similar between responders and non-responders.

**Table 2:** Comparison of responders (persons who returned the Health Risk Appraisal for Older Persons (HRA-O) questionnaire) with non-responders (persons who did not return the HRA-O questionnaire) at the three study sites

**Baseline characteristics**	**London (U.K.)**			**Hamburg (Germany)**			**Solothurn (Switzerland)**		
	**Responders**	**Non-responders**	**P-value**	**Responders**	**Non-responders**	**P-value**	**Responders**	**Non-responders**	**P-value**

Age (years)	74.7 ± 6.3 (1090)	74.8 ± 6.7 (150)	0.70	71.5 ± 7.6 (804)	75.5 ± 7.8 (74)	0.002	74.5 ± 5.8 (748)	74.4 ± 6.0 (126)	0.87
Female gender	55.0% (599/1090)	58.0% (87/150)	0.71	60.9% (490/804)	67.6% (50/74)	0.67	56.7% (424/748)	57.9% (73/126)	0.87
Fair/poor self-perceived health	23.1% (252/1090)	34.7% (52/150)	0.004	36.3% (292/804)	63.5% (47/74)	<.0001	19.0% (142/748)	25.4% (32/126)	0.10
≥ 1 hospital admission over past 12 months	13.6% (148/1090)	16.7% (25/150)	0.52	21.6% (174/804)	16.2% (12/74)	0.09	20.5% (153/748)	16.7% (21/126)	0.23
> 6 physician visits over past 12 months	20.5% (223/1090)	26.7% (40/150)	0.16	50.1% (403/804)	47.3% (35/74)	0.15	23.3% (174/748)	28.6% (36/126)	0.06
No available caregiver if needed	17.2% (187/1090)	20.7% (31/150)	0.36	16.7% (134/804)	29.7% (22/74)	0.07	10.0% (75/748)	8.7% (11/126)	0.53

Pra score	0.27 ± 0.11 (1090)	0.28 ± 0.12 (150)	0.36	0.30 ± 0.11 (804)	0.30 ± 0.12 (74)	0.71	0.29 ± 0.11 (748)	0.29 ± 0.10 (126)	0.86

Values are percentages (nominator/denominator) or means ± standard deviations (denominator).

P-values based on multivariable logistic regression models with covariate pool consisting of the individual Pra items.

Pra score: Probability of repeated admissions; higher scores denote higher risk for hospital admission and other adverse outcomes. for definition see Methods section.

### Feed-back to the HRA-O questionnaire

Acceptance of the HRA-O questionnaire was high, with more than 85% of persons rating comprehension and completion of the questionnaire as easy or very easy. Tables 3 and 4 list the participants' feed-back to the HRA-O questionnaire according to participants' age and educational level at the three study sites. As shown in Table 3, a significantly higher proportion of the over 75-year old persons had difficulties with the questionnaire, as compared to younger persons. Similarly, persons with a low level of education had more difficulty comprehending or answering the questionnaire as compared to persons with a higher level of education (Table 4). However, even among subgroups reporting greater difficulty in using the questionnaire, the proportion of older persons rating the questionnaire as difficult was less than 20%.

**Table 3:** Participants' feedback to the HRA-O questionnaire at the three study sites, according to participants' age

	**London (U.K.)**		**Hamburg (Germany)**		**Solothurn (Switzerland)**	
	**<75 years**	**≥75 years**	**<75 years**	**≥75 years**	**<75 years**	**≥75 years**

Comprehension of questions/instructions – % somewhat difficult/very difficult	5.9% (35/598)	11.5% (55/477)**	4.3% (23/535)	6.3% (16/253)	9.2% (38/412)	17.8% (54/304)***
Completion of questionnaire – % somewhat difficult/very difficult	5.9% (35/589)	9.3% (43/464)*	5.0% (25/496)	8.0% (19/237)	7.5% (29/388)	15.8% (44/279)***
Use of assistance for completing questionnaire – % with assistance	8.6% (51/593)	14.6% (69/471)**	5.2% (27/521)	13.1% (32/245)***	16.4% (67/409)	28.6% (88/308)***
Perceived length of questionnaire – % too long	30.9% (183/593)	35.9% (169/471)	28.2% (148/525)	31.9% (76/238)	54.7% (222/406)	58.4% (180/308)
Time for completion – min. (± SD)	42.6 ± 29.3 (589)	56.2 ± 53.0 (464)***	58.8 ± 27.7 (518)	67.6 ± 31.4 (235)***	73.1 ± 39.6 (388)	83.2 ± 48.2 (282)**
Dislike certain sections of questionnaire – % agreeing	4.9% (28/575)	5.9% (27/461)	9.1% (46/507)	6.4% (14/218)	11.8% (44/373)	13.7% (38/277)
Questionnaire should include additional domains – % agreeing	19.5% (112/573)	14.0% (60/429)*	21.0% (102/485)	15.9% (32/201)	5.4% (20/368)	4.5% (12/269)

HRA-O: Health Risk Appraisal for Older Persons

Values are percentages (nominator/denominator) or means ± standard deviations (denominator).

Denominators vary due to missing individual answers.

* denotes .01 <= P < .05; ** denotes .001 <= P < .01; *** denotes P < .001; P-values are based on Fisher's exact test (binary variables) and t-test (continuous variables).

**Table 4:** Participants' feedback to the HRA-O questionnaire at the three study sites, according to participants' educational level (low vs. medium/high)

	**London (U.K.)**		**Hamburg (Germany)**		**Solothurn (Switzerland)**	
	**low**	**medium/high**	**low**	**medium/high**	**low**	**medium/high**

Comprehension of questions/instructions – % somewhat difficult/very difficult	8.9% (57/642)	7.5% (32/426)	7.9% (11/140)	3.8% (23/601)	17.1% (54/316)	8.8% (31/352)**
Completion of questionnaire – % somewhat difficult/very difficult	8.0% (50/626)	6.4% (27/420)	8.5% (11/129)	5.3% (30/563)	15.4% (46/299)	7.0% (23/329)***
Use of assistance for completing questionnaire – % with assistance	13.1% (83/636)	8.5% (36/423)*	17.6% (24/136)	5.5% (32/583)***	31.4% (100/318)	14.5% (51/351)***
Perceived length of questionnaire – % too long	35.7% (227/636)	28.7% (121/421)*	33.3% (46/138)	27.6% (160/580)	60.3% (190/315)	53.0% (186/351)
Time for completion – min. (± SD)	51.3 ± 46.2 (627)	44.6 ± 34.6 (419)**	65.0 ± 31.6 (131)	60.8 ± 28.2 (576)	78.9 ± 46.9 (297)	75.5 ± 39.3 (330)
Dislike certain sections of questionnaire – % agreeing	4.2% (26/612)	6.8% (28/411)	9.7% (12/124)	7.8% (44/563)	11.5% (33/286)	12.9% (41/317)
Questionnaire should include additional domains – % agreeing	13.4% (79/591)	22.5% (90/400)***	14.4% (17/118)	20.7% (111/535)	1.8% (5/281)	7.4% (23/311)**

HRA-O: Health Risk Appraisal for Older Persons

Values are percentages (nominator/denominator) or means ± standard deviations (denominator).

Denominators vary due to missing individual answers.

* denotes .01 <= P < .05; ** denotes .001 <= P < .01; *** denotes P < .001; P-values are based on Fisher's exact test (binary variables) and t-test (continuous variables).

The proportion of persons using assistance for completing the questionnaire ranged from 5 to 31% percent according to subgroup (age or educational level) and study site. Self-reported time needed for completing the questionnaires varied between study sites and participant age. Persons in Solothurn needed more time to complete the questionnaire compared to persons in London and Hamburg. Those over 75 needed significantly more time to complete the questionnaire compared to younger persons in all sites. Many study participants felt the questionnaire was too long. On the other hand, there was a notable minority of participants suggesting that additional domains should be added to the questionnaire.

### Prevalence of identified risks and problems

Table 5 lists the prevalence of risks and problems identified among study participants at the three study sites. Prevalence rates of identified risks or problems were between 2 and 91% for the 19 domains covered in the HRA-O. For some risks there were notable differences in prevalence rates between sites (e.g., consumption of high fat food, preventative care use, marginal family ties), indicating that regional factors are associated with risks. Overall, at each site the HRA-O questionnaire revealed relatively high (>10%) prevalence rates of most potentially modifiable risk factors for functional decline.

**Table 5:** Prevalence of risks and problems identified with the HRA-O questionnaire at the three study sites

**Domain**	**Definition of problem/risk**	**London (U.K.)**	**Hamburg (Germany)**	**Solothurn (Switzerland)**
Accident prevention	Driving without using seat belt	16.6% (174/1051)	4.8% (37/765)	12.7% (90/707)
Alcohol use	Possible hazardous alcohol use	20.4% (219/1071)	18.8% (133/706)	14.4% (85/591)
Falls	History of repeated falls in previous 12 months	10.6% (111/1048)	7.5% (58/772)	7.2% (50/691)
	Self-reported limitation of activities due to fear of falling	21.6% (230/1064)	24.1% (189/785)	23.5% (167/711)
Functional status	Difficulty in ≥1 BADL item	4.0% (43/1076)	10.4% (82/790)	6.4% (47/730)
	Difficulty/need for human assistance in ≥2 IADL item	16.7% (178/1063)	23.2% (182/785)	19.1% (135/708)
	Changed way of doing an activity	51.9% (544/1048)	46.7% (362/775)	51.6% (366/709)
	Decreased frequency of activity	36.8% (379/1029)	37.5% (285/760)	37.4% (262/700)
Health status	Moderate or fair self-perceived health status	22.1% (239/1080)	29.9% (238/796)	15.9% (116/730)
Hearing	Impaired hearing	20.7% (206/994)	20.4% (155/759)	28.5% (178/624)
	No hearing check-up in previous year	84.6% (908/1073)	63.9% (508/795)	66.2% (473/715)
Incontinence	Urinary incontinence on > 5 days during the last year	10.7% (111/1042)	27.2% (210/772)	20.6% (144/698)
Medication use	Use of ≥4 medications	34.2% (361/1056)	44.3% (332/749)	30.4% (200/657)
	Use of long-acting benzodiazepine or amitriptyline	5.6% (59/1053)	7.6% (58/768)	7.5% (54/719)
	Medication side effect	11.9% (123/1030)	15.1% (114/755)	9.8% (64/652)
	Difficulties with medication compliance	9.4% (90/961)	8.0% (53/660)	5.9% (33/564)
Medical History	Presence of three or more chronic condition(s)	33.4% (354/1059)	52.2% (396/758)	39.5% (279/707)
Memory	Memory problems	10.2% (107/1053)	5.2% (41/781)	6.6% (46/701)
Mood	Depressive mood	14.3% (155/1085)	24.1% (191/792)	14.4% (105/731)
Nutrition	Consumption of >2 high fat food items per day	76.1% (788/1035)	35.1% (258/735)	55.7% (354/635)
	Consumption of <5 fruit/fibre items per day	61.1% (635/1039)	81.2% (608/749)	74.8% (489/654)
	Body mass index <20	4.8% (49/1030)	2.3% (18/787)	2.0% (14/709)
	Body mass index ≥27	32.9% (339/1030)	41.0% (323/787)	52.9% (375/709)
	Loss of weight	3.4% (36/1069)	4.2% (33/795)	4.8% (35/734)
Oral Health	Oral health problem	43.9% (463/1054)	28.5% (224/787)	27.1% (188/694)
	No dental check in previous year	25.9% (279/1077)	17.4% (139/797)	42.5% (306/720)
Physical activity	Less than 5 times/week moderate or strenuous activity	90.7% (933/1029)	80.1% (595/743)	88.4% (524/593)
Preventative care	No blood pressure check in previous year	17.1% (186/1087)	2.5% (20/792)	4.8% (35/734)
	Elevated self-reported blood pressure	67.5% (166/246)	61.0% (383/628)	58.4% (261/447)
	No mammography in previous 2 years (age < 70)	61.0% (94/154)	not available	70.6% (72/102)
	No cervical smear in previous 3 years	89.7% (525/585)	36.8% (178/484)	60.7% (244/402)
	No cholesterol measurement in previous 5 years (age < 75)	43.2% (261/604)	6.0% (32/534)	24.9% (99/397)
	Elevated self-reported cholesterol (age < 75)	8.7% (8/92)	40.3% (94/233)	15.2% (5/33)
	No faecal occult blood test in previous year (age < 80)	93.0% (796/856)	35.0% (233/665)	68.5% (395/577)
	No blood glucose measurement in previous 3 years	78.7% (852/1082)	10.8% (85/784)	24.7% (172/695)
	No influenza vaccination in previous year	18.2% (198/1087)	40.7% (323/794)	53.9% (395/733)
	No pneumococcal vaccination (ever)	78.8% (853/1083)	89.7% (703/784)	91.3% (639/700)
Pain	Presence of moderate to severe pain	27.9% (291/1044)	37.0% (282/762)	24.9% (166/667)
Social factors	Low level of emotional support	10.6% (114/1076)	8.8% (69/784)	9.4% (64/681)
	High risk of social isolation	14.1% (152/1076)	19.1% (150/784)	9.7% (66/681)
	Marginal family ties	14.4% (155/1076)	18.1% (142/784)	6.6% (45/681)
	Marginal friendship ties	17.9% (193/1076)	20.8% (163/784)	18.5% (126/681)
	No participation in groups	32.2% (347/1077)	37.9% (301/795)	20.9% (149/713)
Tobacco use	Current tobacco use	11.2% (114/1021)	13.1% (97/739)	13.3% (86/645)
Vision	Problem in ≥1 vision sub-domain	16.5% (169/1026)	16.2% (125/770)	13.7% (93/681)
	No vision check-up in previous year	34.1% (369/1081)	28.3% (225/795)	38.3% (280/732)

HRA-O: Health Risk Appraisal for Older Persons

Values are percentages (nominator/denominator).

Denominators vary due to missing individual answers.

For definition of variables, see Table 1.

### Self-reported reasons for suboptimal health behaviour and preventative care use

Table 6 lists participants' intention to change health behaviour and self-reported reasons for suboptimal health behaviour or use of preventative services. With regard to physical activity (level of physical activity within the next month or the next 6 months) and nutrition, only a small minority (5.4 percent or less) declare that they plan to change food intake within the next month or the next 6 months. The most frequently reported reason for not changing level of physical activity and nutrition intake is the self-perception of optimal health behaviour despite evidence for suboptimal health behaviour. For example, In London, among 933 persons with a low level of physical activity, 338 (36.2%) reported that they did not increase their level of physical activity because they thought they already exercised frequently and regularly. With regard to tobacco use, this was different. One third to almost one half of persons using tobacco report that they plan to quit within the next month or the next 6 months.

**Table 6:** Intention to change health behaviour and self-reported reasons for not changing health behaviour/preventative care use identified with the HRA-O questionnaire at the three study sites

**Category**	**Self reported answer category**	**London (U.K.)**	**Hamburg (Germany)**	**Solothurn (Switzerland)**
Intention to increase physical activity	Plans to take steps in next month	2.5% (21/853)	1.0% (5/507)	1.6% (7/426)
	Plans to take steps in the next 6 months	2.9% (25/853)	0.6% (3/507)	0.2% (1/426)
Reasons for not increasing physical acitivity	I already exercise frequently and regularly	36.2% (338/933)	47.7% (284/595)	63.2% (331/524)
	I have a physical limitation	22.0% (205/933)	14.3% (85/595)	6.7% (35/524)
	I don't have time/don't get around it	18.8% (175/933)	6.1% (36/595)	3.8% (20/524)
	I have pain with physical activity	18.0% (168/933)	25.7% (153/595)	16.8% (88/524)
	I have an illness limiting my physical activity	11.5% (107/933)	25.9% (154/595)	13.2% (69/524)
	I don't have anyone to exercise with	5.8% (54/933)	8.4% (50/595)	4.4% (23/524)
	There is nowhere to exercise	1.9% (18/933)	8.7% (52/595)	4.8% (25/524)
Intention to decrease high fat intake	Plans to take steps in next month	2.1% (16/765)	2.8% (7/253)	0.9% (3/333)
	Plans to take steps in the next 6 months	1.6% (12/765)	0.8% (2/253)	0.9% (3/333)
Reason for not decreasing high fat intake	I already minimise fat intake	75.4% (594/788)	70.9% (183/258)	67.5% (239/354)
	I like the taste of high-fat foods	19.5% (154/788)	19.4% (50/258)	9.3% (33/354)
	I don't think it's important to eat less fat	5.3% (42/788)	11.6% (30/258)	13.6% (48/354)
	Trouble to shop/prepare low-fat foods	2.4% (19/788)	5.4% (14/258)	7.6% (27/354)
Intention to increase fruit/fibre intake	Plans to take steps in next month	0.8% (5/623)	0.7% (4/596)	0.2% (1/470)
	Plans to take steps in the next 6 months	1.0% (6/623)	0.2% (1/596)	0.2% (1/470)
Reason for not increasing low fruit/fibre intake	I already eat plenty of fruits/vegetables	87.9% (558/635)	91.0% (553/608)	93.7% (458/489)
Intention to change current tobacco use	Plans to quit smoking in next month	16.7% (14/84)	16.0% (12/75)	22.6% (12/53)
	Plans to quit smoking in next 6 months	28.6% (24/84)	17.3% (13/75)	18.9% (10/53)
Reason for not using preventative services	My general practitioner never recommended it	50.5% (548/1086)	22.2% (173/778)	16.0% (117/731)
	I've never thought about it	21.5% (234/1086)	12.3% (96/778)	10.9% (80/731)
	I have no need to; I have no health problems	17.5% (190/1086)	17.0% (132/778)	20.2% (148/731)
	I have already had these preventative services	15.7% (171/1086)	37.1% (289/778)	39.8% (291/731)

HRA-O: Health Risk Appraisal for Older Persons, values are percentages (nominator/denominator).

Denominators are persons at risk for the selected health behaviour or preventative care use (e.g., intention to decrease high fat intake among persons with high fat intake, as defined in Table 5). Answers are for predefined categories. Multiple answer were allowed for reasons of sub-optimal health behaviour; only reasons given by ≥5% of persons in at least one study site are listed, and listed according to the rank order in London.

The self-reported reasons for not using all preventative care services recommended to older persons varied by site. In London, more than 50% of the participants stated that their general practitioner had never recommended it. In Solothurn and Hamburg, the most frequently given reason was "I have already had these preventative services." Other reasons, such as cost or lack of time were given by less than 5 percent of participants at all sites.

## Discussion

To our knowledge this is the first HRA instrument for use in older persons that has been developed and evaluated outside North America for international use. Base-line data from its use in the PRO-AGE multi-centre trial confirm that the HRA-O questionnaire is feasible in this population, including those at advanced age and with lower educational levels. In addition, the HRA-O questionnaire identifies a large number of potentially modifiable risks for functional decline and related problems. Participants' intention to change, and self-reported reasons for not changing health behaviour suggested that for some risk factors participants were in a pre-contemplation phase [27], having no short- or medium-term plans for changing health behaviour, and many perceived their health behaviour or preventative care uptake as optimal, despite indications of deficits according to the HRA-O based evaluation.

There are some limitations. This study might overestimate the response rate to the HRA-O questionnaire because only those participants who had given informed consent to participate in the study were sent the HRA-O questionnaire. This limitation cannot be avoided in the context of a controlled trial. Despite this limitation, the response rate of >80% for a multidimensional questionnaire, without a reminder system, is remarkable and underlines its practicability. One likely explanation of the high response rate was the contribution of the general practitioner's relationship with his/her patients.

Second, although acceptability of the instrument would certainly differ in populations with a very low level of education (in this project, most persons classified as having a low level of education had had 9 years of education), it is likely that the instrument can be used at other sites as well. The three study sites represented here include urban and rural regions, different languages, different health care systems, and persons with a broad range of socioeconomic characteristics.

Third, the prevalence rates found in this study may not be representative of the population of community-dwelling older persons in these regions. Participants were selected according to practice registration and eligibility criteria, and persons not interested in participating in the study were excluded. Nevertheless, comparison of participant characteristics with available national data reveals similarities, suggesting that findings of this study give appropriate estimates for non-disabled non-institutionalised older persons.

## Conclusion

This study has implications for practice and research. HRA-O has multiple advantages, compared with other HRA tools for older persons. For one, the present HRA-O shares the distinguishing features of HRA-E, as described earlier. In addition, this study gave evidence that HRA-O has additional unique benefits: HRA-O has high acceptance rates and good feasibility in community-dwelling older persons at different sites, and HRA-O has proven to be functional in a multilingual mode.

At the present time, many intervention programs addressing health promotion and prevention have used alternative strategies requiring a large amount of professional time without a self-administered component. For example, most programs of preventive home visits start with an approximately two-hour multidimensional evaluation of older persons by a health professional [38]. Other programs use a self-administered survey approach, but are limited to a brief questionnaire focussing on general aspects of health risks and do not address all potential risk domains with domain-specific screening instruments [1].

There is potential for further development. First, with additional data and analyses from the PRO-AGE study, a further update of the HRA-O is currently under way. Second, in the UK, the Department of Health is currently funding a study to identify social aspects that could be added to HRA-O [39]. Third, in the future, it might be possible to give quantitative estimates of individual risks for functional decline, and the potential impact of risk factor modification. The HRA-O instrument combined with specific interventions might be a promising tool for individualised health promotion and prevention programs in older persons.

## Competing interests

The author(s) declare that they have no competing interests.

## Authors' contributions

All authors are members of the PRO-AGE project group and participated in the conceptualisation and implementation of the study. HM and UD were the administrative coordinators of PRO-AGE project, AS was the technical/scientific coordinator of the project. AS, JB, CS, and HM developed the study plan. KK, DH, SI, CS implemented the London (U.K.) trial; UD, JA, WR, HM implemented the Hamburg (Germany) trial; AS was responsible for the implementation of the study in Solothurn (Switzerland). GG, ME, KI, and AS performed the central data management and data analysis. JH and JB was responsible for HRA-O development in the United States. JB was involved as senior consultant to the project, and contributed to the trial design, data analysis, and data interpretation. AS and KK developed the first version of this manuscript. All authors contributed to the present manuscript.

## Pre-publication history

The pre-publication history for this paper can be accessed here:



## Supplementary Material

Additional File 1 Older Persons Health Profile Questionnaire, version 2000, U.K. English version. Health Risk Appraisal (HRA-O) Questionnaire U.K. (non-printable PDF document, 34 pages). Click here for file

## References

[B1] Rand Corporation (2000). Health Risk Appraisals and Medicare. Evidence report and evidence-based recommendations.

[B2] Breslow L, Beck JC, Morgenstern H, Fielding JE, Moore AA, Carmel M, Higa J (1997). Development of a health risk appraisal for the elderly (HRA-E). Am J Health Promot.

[B3] Stuck AE, Kharicha K, Dapp U, Anders J, Von Renteln-Kruse W, Meier-Baumgartner HP, Iliffe S, Harari D, Bachmann MD, Egger M, Gillmann G, Beck JC, Swift CG (2007). The PRO-AGE study: An international randomised controlled study of health risk appraisal for older persons based in general practice. BMC Med Res Methodol.

[B4] Stuck AE, Walthert J, Nikolaus T, Büla CJ, Hohmann C, Beck JC (1999). Risk factors for functional status decline in community-dwelling elderly people: a systematic literature review. Soc Sci Med.

[B5] Peter-Wüest I, Stuck AE, Dapp U, Nikolaus T, Goetz SM, Gillmann G, Beck JC (2000). A new multidimensional instrument for preventive in-home assessments in older people: results of a pilot test. Z Gerontol Geriat.

[B6] Goetz SM, Stuck AE, Hirschi A, Gillmann G, Dapp U, Nikolaus T, Minder CE, Beck JC (2001). Test-retest reliability of a newly developed German language instrument for multidimensional geriatric assessment. Z Geront Geriat.

[B7] Goetz SM, Stuck AE, Hirschi A, Gillmann G, Dapp U, Minder CE, Beck JC (2000). A new multidimensional assessment instrument in German for prevention in older persons: Comparison of the self-administered with the interviewer-administered version. Z Soz-Präventivmedizin.

[B8] Stuck AE, Elkuch P, Dapp U, Anders J, Iliffe S, Swift CG (2002). Feasibility and yield of a self-administered questionnaire for health risk appraisal in older people in three European countries. Age Ageing.

[B9] Iliffe S, Kharicha K, Harari D, Swift C, Stuck AE (2005). Health risk appraisal for older people in general practice using an expert system: a pilot study. Health & Social Care in the Community.

[B10] Ludwig R (2002). Preventive health care use in older people in Switzerland. Thesis, University of Bern.

[B11] Atchison KA, Dolan TA (1990). Development of the Geriatric Oral Health Assessment Index. J Dent Educ.

[B12] Ferrell BA, Stein WM, Beck JB (2000). The Geriatric Pain Measure: Validity, reliability and factor analysis. J Am Geriatr Soc.

[B13] Fried LP, Bandeen-Roche K, Chaves PH, Johnson BA (2000). Preclinical mobility disability predicts incident mobility disability in older women. J Gerontol A Biol Sci Med Sci.

[B14] Beers MH (1997). Explicit criteria for determining potentially inappropriate medication use by the elderly. Arch Intern Med.

[B15] Babor TF, de la Fuente JR, Saunders J, Grant M (1992). AUDIT – The Alcohol Use Disorders Identification Test: guidelines for use in primary health care.

[B16] Berkman LF, Syme SL (1979). Social networks, host resistance, and mortality: A nine-year follow-up study of Alameda County residents. Am J Epidemiol.

[B17] Diokno AC, Brock BM, Brown MB, Herzog AR (1986). Prevalence of urinary incontinence and other urological symptoms in the noninstitutionalized elderly. J Urol.

[B18] Human Population Laboratory (1965). Health and Ways of Living. Human Population Laboratory (HPL), Men's Form.

[B19] Katz S, Ford AB, Moskowitz RW, Jackson BA, Jaffe MW (1963). Studies of illness in the aged. The index of ADL: A standardized measure of biological and psychosocial function. JAMA.

[B20] Kelsey JL, Browner WS, Seeley DG, Nevitt MC, Cummings SR (1992). Risk factors for fractures of the distal forearm and proximal humerus. Am J Epidemiol.

[B21] Kreuter MW, Strecher VJ (1995). Changing inaccurate perceptions of health risk: Results from a randomized trial. Health Psychol.

[B22] Lawton MP, Brody EM (1969). Assessment of older people: Self-maintaining instrumental activities of daily living. Gerontologist.

[B23] Lichtenstein MJ, Bess FH, Logan SA (1988). Validation of screening tools for identifying hearing-impaired elderly in primary care. JAMA.

[B24] Lubben JE (1988). Assessing social networks among elderly populations. Fam Comm Health.

[B25] Mangione CM, Lee PP, Pitts J, Gutierrez P, Berry S, Hays RD (1998). Psychometric properties of the National Eye Institute Visual Function Questionnaire (NEI-VFQ). Arch Ophthalmol.

[B26] National Center for Chronic Disease Prevention and Health Promotion (1993). Behavioral Risk Factor Survey.

[B27] Prochaska JO, DiClemente CC, Norcoss JC (1992). In search of how people change: applications to addictive behaviours. Am Psychol.

[B28] Riege WH (1982). Self-report and tests of memory aging. Clin Gerontol.

[B29] Sherbourne CD, Stewart AL (1991). The MOS social support survey. Soc Sci Med.

[B30] Stewart AL, Hays RD, Ware JE (1988). The MOS short-form general health survey. Reliability and validity in a patient population. Med Care.

[B31] Stoy DB, Curtis RC, Dameworth KS, Dowdy AA, Hegland J, Levin JA, Sousoulas BG (1995). The successful recruitment of elderly black subjects in a clinical trial: the CRISP experience. Cholesterol Reduction in Seniors Program. J Natl Med Assoc.

[B32] Tinetti ME, Speechley M, Ginter SF (1988). Risk factors for falls among elderly persons living in the community. N Engl J Med.

[B33] US Preventive Health Services Task Force (1996). Guide to clinical preventive services.

[B34] Washburn RA, Smith KW, Jette AM, Janney CA (1993). The Physical Activity Scale for the Elderly (PASE): development and evaluation. J Clin Epidemiol.

[B35] Wasson J, Nierenberg D, Landgraf J (1992). The effect of a patient questionnaire on drug-related symptoms in elderly outpatients. Ann Rev Geriatr & Gerontol.

[B36] Boult C, Dowd B, McCaffrey D, Boult L, Hernandez R, Krulewitch H (1993). Screening elders for risk of hospital admission. J Am Geriatr Soc.

[B37] SAS Institute Inc (2004). SAS/STAT^® ^9.1 User's Guide.

[B38] Stuck AE, Egger M, Hammer A, Minder CE, Beck JC (2002). Home visits to prevent nursing home admission and functional decline in elderly people: Systematic review and meta-regression analysis. JAMA.

[B39] Iliffe S, Kharicha K, Goodman C, Harari D, Swift C, Manthorpe J (2005). Smarter working in primary care. Quality in Ageing.

