# Supplementary material for: Development, feasibility and performance of a health risk appraisal questionnaire for older persons
Source: BMC Med Res Methodol. 2007 Jan 11;7:1. doi: 10.1186/1471-2288-7-1 (PMC1783663; doi:10.1186/1471-2288-7-1)
Supplement: Additional File 1 — Older Persons Health Profile Questionnaire, version 2000, U.K. English version. Health Risk Appraisal (HRA-O) Questionnaire U.K. (non-printable PDF document, 34 pages). [file 1471-2288-7-1-S1.pdf]

Older Persons Health Profile Questionnaire

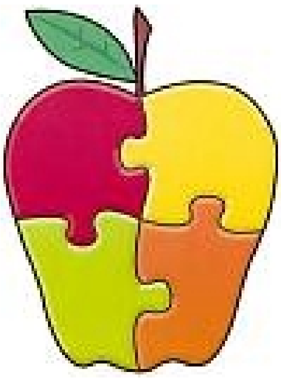

Please check whether your personal details are correct. If there are mistakes please correct them on the following lines. It would be helpful for us to have your daytime home phone number.

not for use without written copyright permission

____

____

____

Please fill out your Older Persons Health Profile Questionnaire to receive your confidential and personal Older Persons Health Profile Report. Your report will give you information on how you are doing in areas of your health – such as nutrition, safety, medication use, and preventive care.

Remember...

- *Read each question carefully, and follow all directions.*
- *Tick boxes as shown here:* [X]
- *Print all written responses clearly, as shown here:* [M][A][R][Y]
- *Please do not write comments in the margins -- these cannot be responded to.*
- *Complete all sections and items.*
- *Do not leave any answers blank, even if your answer is "No".*
- *Be careful to fill out each page.*

© 2000 This questionnaire is protected by copyright.

Medical History

Here is a list of medical conditions that usually last for some time.

Has a doctor ever told you that you have…		No	Yes
1.	... High blood pressure?	☐ 1	☐ 2
2.	... Coronary heart disease or heart attack?	☐ 1	☐ 2
3.	... Heart failure?	☐ 1	☐ 2
4.	... Irregular heart beat?	☐ 1	☐ 2
5.	... Stroke?	☐ 1	☐ 2
6.	... Chronic bronchitis or emphysema?	☐ 1	☐ 2
7.	... Asthma?	☐ 1	☐ 2
8.	... Arthritis or rheumatism?	☐ 1	☐ 2
9.	... Osteoporosis?	☐ 1	☐ 2
10.	... Diabetes?	☐ 1	☐ 2
11.	... Depression?	☐ 1	☐ 2
12.	... Emotional [illegible] mental illness other than depression?	☐ 1	☐ 2
13.	... Glaucoma?	☐ 1	☐ 2
14.	... Irreversible / untreatable retinal disease?	☐ 1	☐ 2
15.	... Cataracts?	☐ 1	☐ 2

not for use without written copyright permission

© 2000 This questionnaire is protected by copyright.

Your Health Measurements

1. What is your height (in feet/inches or meters/centimeters, without shoes)?

   feet inches or m cm

2. What is your weight (in stones/pounds or kilograms, wearing light clothing without shoes)?

   stones pounds or kg

3. Have you lost 5 kg (a stone) or more <u>over the past six months</u> without trying to do so?

   ☐1 No
   ☐2 Yes

4. Have you gained 5 kg (a stone) or more <u>over the past six months</u> without trying to do so?

   ☐1 No
   ☐2 Yes

5. What is your blood pressure?

   (Systolic) (Diastolic)

   ☐1 Don't know/not sure

6. What is your total cholesterol level? .

   ☐1 Don't know/not sure

not for use without written copyright permission

© 2000 This questionnaire is protected by copyright.

Medications/Prescriptions

	Indicate whether or not you are now taking:	No	Yes
1.	Medicine for heart conditions	☐1	☐2
2.	Medicine for high blood pressure	☐1	☐2
3.	Medicine for high cholesterol	☐1	☐2
4.	Medicine for diabetes	☐1	☐2
5.	Medicine to help you fall asleep (3 or more times per week)	☐1	☐2
6.	Medicine for anxiety (nerves or nervousness)	☐1	☐2
7.	Medicine for depression	☐1	☐2
8.	Medicine to replace female hormones (Women only)	☐1	☐2
9.	Medicine for arthritis or pain in the joints (3 or more times per week)	☐1	☐2
10.	Aspirin regularly to prevent heart attack or stroke	☐1	☐2

not for use without written copyright permission

The following questions ask, by name, about specific medications you may take.

	**During the last 4 weeks, have you taken any of these medications at least once?**	No	Yes
11.	Valium, Diazepam, Nitrazepam or Mogadon?	☐1	☐2
12.	Chlordiazepoxide or Librium?	☐1	☐2
13.	Amitriptyline?	☐1	☐2

14. How many different medications are you currently taking? *Include those to which you answered "Yes" above*

(Write in "0" if you are taking no medications.)

☐☐ Number of prescription medications

☐☐ Number of over-the-counter medications (for example hay fever remedies or pain killers)

☐☐ Number of herbal remedies (for example St. John's Wort)

15. Do you think that you are having any symptoms due to your medications?
☐1 No
☐2 Yes

© 2000 This questionnaire is protected by copyright.

16. Which of the following keep you from taking your medication as prescribed?
Please tick all that apply.

a. ☐1 I have not been prescribed any medications
b. ☐1 I take my medications as prescribed
c. ☐1 I forget or don't think about it
d. ☐1 It’s not that important
e. ☐1 I don’t have time/don't get around to it
f. ☐1 Cost
g. ☐1 Side effects
h. ☐1 Other

not for use without written copyright permission

© 2000 This questionnaire is protected by copyright.

Signs & Symptoms

These questions are about symptoms you had during the last month.

**Indicate how frequently <u>during the last month</u> you have been bothered by the following symptoms for any reason:**		**Never**	**Seldom or Some-times**	**Often or Always**
1.	Fever	☐1	☐3	☐5
2.	Unexplained tiredness/weakness	☐1	☐3	☐5
3.	Inability to think well	☐1	☐3	☐5
4.	Nightmares	☐1	☐3	☐5
5.	Depression/feeling low	☐1	☐3	☐5
6.	Anxiety/agitation	☐1	☐3	☐5
7.	Blurred eyesight *(despite wearing glasses or contact lenses)*	☐1	☐3	☐5
8.	Nasal stuffiness	☐1	☐3	☐5
9.	Abnormal taste	☐1	☐3	☐5
10.	Dry mouth	☐1	☐3	☐5
11.	Sleepiness other than at bedtime	☐1	☐3	☐5
12.	Shortness of breath at night	☐1	☐3	☐5
13.	Shortness of breath with little exercise	☐1	☐3	☐5
14.	Leg swelling	☐1	☐3	☐5
15.	Heart pounding or irregular heart beat	☐1	☐3	☐5
16.	Dizziness when standing up	☐1	☐3	☐5
17.	Fainting spells	☐1	☐3	☐5
18.	Chest pain	☐1	☐3	☐5
19.	Cough	☐1	☐3	☐5
20.	Constipation	☐1	☐3	☐5
21.	Loose stools (diarrhoea)	☐1	☐3	☐5

not for use without written copyright permission

© 2000 This questionnaire is protected by copyright.

Indicate how frequently <u>during the last month</u> you have been bothered by the following symptoms for any reason:		Never	Seldom or Some-times	Often or Always
22.	Sickness in stomach (nausea)	☐1	☐3	☐5
23.	Abdominal pain	☐1	☐3	☐5
24.	Heartburn	☐1	☐3	☐5
25.	Inability to pass urine	☐1	☐3	☐5
26.	Urinating at night more than twice	☐1	☐3	☐5
27.	Excessive urination	☐1	☐3	☐5
28.	Skin rash	☐1	☐3	☐5
29.	Skin itching	☐1	☐3	☐5
30.	Breast tenderness (for men and women)	☐1	☐3	☐5
31.	Leg ache when exercising	☐1	☐3	☐5
32.	Unexplained muscle aches	☐1	☐3	☐5
33.	Sexual problems	☐1	☐3	☐5
34.	Headache	☐1	☐3	☐5
35.	Cold or painful hands or feet	☐1	☐3	☐5

not for use without written copyright permission

Bladder control

Loss of bladder control becomes more common with age; however, in many cases, bladder control can be improved.

36a. <u>In the last year</u>, have you had leakage of urine and got wet?

☐1 No → *Skip to page 8 (Pain)*

☐2 Yes ↓

36b. ***If Yes***: Have you had leakage of urine on <u>at least 6 separate days</u>?

☐1 No

☐2 Yes

© 2000 This questionnaire is protected by copyright.

Pain

1. Over the **last 4 weeks**, have you had any pain?
   ☐1 No ➔ *Skip to page 10 (Oral Health)*
   ☐2 Yes

*The next 2 questions ask about your experience of pain, based on a scale from zero to ten, with "**0**" meaning "no pain" and "**10**" meaning "the worst pain you can imagine".*

2. How severe is your pain ***today***?

0	1	2	3	4	5	6	7	8	9	10
☐0	☐1	☐2	☐3	☐4	☐5	☐6	☐7	☐8	☐9	☐10

no pain worst pain

3. In the ***last 7 days***, how severe has your pain been *on* ***average***?

0	1	2	3	4	5	6	7	8	9	10
☐0	☐1	☐2	☐3	☐4	☐5	☐6	☐7	☐8	☐9	☐10

no pain worst pain

The next questions ask about [illegible] that can occur with different activities. Choose the answer that comes closest to being accurate for you.

not for use without written copyright permission

		No	Yes
4.	Do you have pain every day?	☐1	☐2
5.	Do you have pain several times a week?	☐1	☐2
6.	Do you have pain that never completely goes away?	☐1	☐2
7.	Do you take medicine for pain 3 or more times per week?	☐1	☐2
8.	Over the last 7 days, has pain caused you to feel sad and depressed?	☐1	☐2

© 2000 This questionnaire is protected by copyright.

Do you currently have pain with the following activities? Or have you stopped doing any of them because of <u>pain</u>?		No	Yes
9.	Bathing or dressing	☐1	☐2
10.	Lifting or carrying groceries	☐1	☐2
11.	Walking 200 yards or less	☐1	☐2
12.	Walking more than 200 yards	☐1	☐2
13.	Climbing only a few steps	☐1	☐2
14.	Climbing more than one flight of stairs	☐1	☐2
15.	Moderate activities *such as moving a heavy table, pushing a vacuum cleaner, bowling, or playing golf*	☐1	☐2
16.	Vigorous activities *such as running, lifting heavy objects, or participating in strenuous sports*	☐1	☐2

**Does <u>pain</u> prevent you from…**		**No**	**Yes**
17.	... travelling or using public transport?	☐1	☐2
18.	... attending religious activities?	☐1	☐2
19.	... enjoying any other social or recreational activities (other than religious services)?	☐1	☐2

not for use without written copyright permission

**Because of <u>pain</u>, …**		**No**	**Yes**
20.	... have you cut down the amount of time you spend on work or other activities?	☐1	☐2
21.	... have you been accomplishing less than you would like to?	☐1	☐2
22.	... have you limited the kind of work or other activities you do?	☐1	☐2
23.	... does the work or activities you do require extra effort?	☐1	☐2
24.	... do you have trouble sleeping?	☐1	☐2
25.	... do you feel fatigued or tired?	☐1	☐2
26.	... do you have to rely on family members or friends for help?	☐1	☐2

© 2000 This questionnaire is protected by copyright.

Oral Health

1. Do you wear a removable set of dentures (either full or partial)?
   - ☐1 No
   - ☐2 Yes

	In the <u>past 3 months</u>, how often …	Never	Seldom	Some-times	Often	Always
2.	… did you limit the kinds or amounts of food you ate because of problems with your teeth or dentures?	☐1	☐2	☐3	☐4	☐5
3.	… did you have trouble biting or chewing any kinds of food, *such as firm meat or apples?*	☐1	☐2	☐3	☐4	☐5
4.	… were you able to swallow **<u>comfortably</u>**?	☐1	☐2	☐3	☐4	☐5
5.	… have your teeth or dentures prevented you from speaking the way you wanted?	☐1	☐2	☐3	☐4	☐5
6.	… were you **<u>able to eat anything</u>** without feeling discomfort?	☐1	☐2	☐3	☐4	☐5
7.	… did you limit contacts with people because of the condition of your teeth or dentures?	☐1	☐2	☐3	☐4	☐5
8.	… were you **<u>pleased or happy</u>** with the look of your teeth and gums or dentures?	☐1	☐2	☐3	☐4	☐5
9.	… did you use medication to relieve pain or discomfort from around your mouth?	☐1	☐2	☐3	☐4	☐5
10.	… were you worried or concerned about the problems with your teeth, gums or dentures?	☐1	☐2	☐3	☐4	☐5
11.	… did you feel nervous or self-conscious because of problems with your teeth, gums or dentures?	☐1	☐2	☐3	☐4	☐5
12.	… did you feel uncomfortable eating in front of people because of problems with your teeth or dentures?	☐1	☐2	☐3	☐4	☐5
13.	… were your teeth or gums sensitive to hot, cold or sweets?	☐1	☐2	☐3	☐4	☐5

not for use without written copyright permission

© 2000 This questionnaire is protected by copyright.

Vision

The following questions address problems which involve your eyesight or feelings that you have about your eyesight condition. After each question, please choose the response that best describes your situation.

1. At the present time, would you say your eyesight using both eyes (*with your glasses or contact lenses, if you wear them*) is:
   - ☐ 1 Excellent → *Skip to page 14 (Hearing)*
   - ☐ 2 Good
   - ☐ 3 Fair
   - ☐ 4 Poor
   - ☐ 5 Very Poor
   - ☐ 6 Completely Blind

2. How much of the time do you worry about your eyesight?
   - ☐ 1 None of the time
   - ☐ 2 A little of the time
   - ☐ 3 Some of the time
   - ☐ 4 Most of the time
   - ☐ 5 All of the time

3. Have you noticed any recent worsening of your eyesight?
   - ☐ 1 No
   - ☐ 2 Yes

not for use without written copyright permission

The next questions are about how much difficulty, if any, you have doing certain activities (wearing your glasses or contact lenses if you use them for that activity).

4. How much difficulty do you have reading ordinary print in newspapers? Would you say you have:
   - ☐ 1 No difficulty at all
   - ☐ 2 A little difficulty
   - ☐ 3 Moderate difficulty
   - ☐ 4 Extreme difficulty
   - ☐ 5 Stopped doing this because of your eyesight
   - ☐ 6 Stopped doing this for other reasons or not interested in doing this

5. How much difficulty do you have doing work or hobbies that require close vision, *such as cooking, sewing, fixing things around the house, or using hand tools*? Would you say, you have:
   - ☐ 1 No difficulty at all
   - ☐ 2 A little difficulty
   - ☐ 3 Moderate difficulty
   - ☐ 4 Extreme difficulty
   - ☐ 5 Stopped doing this because of your eyesight
   - ☐ 6 Stopped doing this for other reasons or not interested in doing this

© 2000 This questionnaire is protected by copyright.

6. Because of your eyesight, how much difficulty do you have going down steps, stairs, or kerbs in dim light or at night?
   - ☐ 1 No difficulty at all
   - ☐ 2 A little difficulty
   - ☐ 3 Moderate difficulty
   - ☐ 4 Extreme difficulty
   - ☐ 5 Stopped doing this because of your eyesight
   - ☐ 6 Stopped doing this for other reasons

7. Because of your eyesight, how much difficulty do you have noticing objects off to the side while you are walking along?
   - ☐ 1 No difficulty at all
   - ☐ 2 A little difficulty
   - ☐ 3 Moderate difficulty
   - ☐ 4 Extreme difficulty
   - ☐ 5 Stopped doing this because of your eyesight
   - ☐ 6 Stopped doing this for other reasons

8. Because of your eyesight, how much difficulty do you have finding something on a crowded shelf up close to you?
   - ☐ 1 No difficulty at all
   - ☐ 2 A little difficulty
   - ☐ 3 Moderate difficulty
   - ☐ 4 Extreme difficulty
   - ☐ 5 Stopped doing this because of your eyesight
   - ☐ 6 Stopped doing this for other reasons

not for use without written copyright permission

9. Are you limited in how long you can work or do other activities because of your eyesight?
   - ☐ 1 None of the time
   - ☐ 2 A little of the time
   - ☐ 3 Some of the time
   - ☐ 4 Most of the time
   - ☐ 5 All of the time

© 2000 This questionnaire is protected by copyright.

The next questions are about car driving.

10a. Are you currently driving, at least once in a while?

- ☐1 Yes → *Skip to question 11 on this page*
- ☐2 No

10b. ***If No:*** Have you never driven a car, or have you given up driving?

- ☐1 Never drove → *Skip to page 14 (Hearing)*
- ☐2 Gave up ↓

10c. Why did you give up driving? Was that mainly because of your eyesight, mainly for some other reason, or because of both your eyesight and other reasons?

- ☐1 Mainly eyesight
- ☐2 Mainly other reasons
- ☐3 Both eyesight and other reasons

} → *Skip to page 14 (Hearing)*

The following question is for current drivers.

11. How much difficulty do you have driving during the daytime in familiar places
Would you say you have:

- ☐1 No difficulty at all
- ☐2 A little difficulty
- ☐3 Moderate difficulty
- ☐4 Extreme difficulty

not for use without written copyright permission

© 2000 This questionnaire is protected by copyright.

Hearing

1. How would you rate your hearing *(with your hearing aid on, if applicable)*?

☐1 Excellent → *Skip to page 15 (Psychosocial & Well Being)*
☐2 Good
☐3 Fair
☐4 Poor
☐5 Very poor
☐6 Deaf

	**Does your hearing (with your hearing aid, if you wear one) cause you ...**	**No**	**Some-times**	**Yes**
2.	... to feel embarrassed when you meet new people?	☐1	☐2	☐3
3.	... to feel frustrated when talking to members of your family?	☐1	☐2	☐3
4.	... to have difficulty when visiting friends, relatives, or neighbours?	☐1	☐2	☐3
5.	... to attend religious services less often than you would like?	☐1	☐2	☐3
6.	... to have arguments with family members?	☐1	☐2	☐3
7.	... to have difficulty when listening to radio or television?	☐1	☐2	☐3
8.	... to have difficulty when in a restaurant with relatives or friends?	☐1	☐2	☐3
9.	... to have difficulty hearing when someone speaks in a whisper?	☐1	☐2	☐3
10.	... to feel handicapped by a hearing problem?	☐1	☐2	☐3
11.	... to limit/hamper your personal or social life?	☐1	☐2	☐3

not for use without written copyright permission

12a. Do you wear a hearing aid?

☐1 No → *Skip to page 15 (Psychosocial & Well Being)*
☐2 Yes

12b. ***If Yes:*** On average, how often do you change the batteries in your hearing aid?

☐1 Every 1-2 weeks or more
☐2 Every 3-4 weeks
☐3 Once a month or less

© 2000 This questionnaire is protected by copyright.

Psychosocial Health & Well Being

*These questions are about how you feel and how things have been with you <u>during the past month</u>. For each question, please give the **ONE** answer that comes closest to the way you have been feeling.*

**How much of the time, <u>during the last month</u>, have you …**		**None of the time**	**A little of the time**	**Some of the time**	**A good bit of the time**	**Most of the time**	**All of the time**
1.	... felt calm and peaceful?	☐1	☐2	☐3	☐4	☐5	☐6
2.	... been a very nervous person?	☐1	☐2	☐3	☐4	☐5	☐6
3.	... felt so down in the dumps that nothing could cheer you up?	☐1	☐2	☐3	☐4	☐5	☐6
4.	... felt downhearted and low?	☐1	☐2	☐3	☐4	☐5	☐6
5.	... been a happy person?	☐1	☐2	☐3	☐4	☐5	☐6

6. If you are feeling down, has there been any special reason for your recent mood?
For example, loss of a loved one, change in living situation, etc.
☐1 No ☐3 Not applicable
☐2 Yes

not for use without written copyright permission

Please select the <u>best</u> response for each of the following questions about your memory:		**Almost never**	**Some-times**	**Often**	**Almost always**
7.	Can you remember what clothes you wore yesterday?	☐1	☐2	☐3	☐4
8.	In a large department store do you usually remember what entrance you used?	☐1	☐2	☐3	☐4
9.	Can you recall where you parked your car or where your lift will pick you up?	☐1	☐2	☐3	☐4
10.	In the dark can you distinguish between your car key and your house key?	☐1	☐2	☐3	☐4
11.	Do you remember where you put your glasses or keys?	☐1	☐2	☐3	☐4
12.	If someone calls you, can you give him or her directions to your house?	☐1	☐2	☐3	☐4
13.	If you watch a TV series that runs for several nights, do you remember what happened in the last episode?	☐1	☐2	☐3	☐4
14.	Can you describe what you did last Sunday afternoon?	☐1	☐2	☐3	☐4
15.	Can you hum a tune after you have heard it several times?	☐1	☐2	☐3	☐4
16.	Could you remember how to put back together a small appliance after you have taken it apart?	☐1	☐2	☐3	☐4

© 2000 This questionnaire is protected by copyright.

Social Network

People sometimes look to others for friendship, assistance, or other types of support.

The following three questions are about your family members, including your spouse, in-laws, and any other relatives.

1. How many relatives or family members do you see or hear from <u>at least once a month</u>? (Note: Include spouse, in-laws, and any other relatives.)
   - ☐1 Nine or more
   - ☐2 Five to eight
   - ☐3 Three or four
   - ☐4 Two
   - ☐5 One
   - ☐6 None

2. How many relatives or family members do you feel close to that you can call on for help? (Note: Include spouse, in-laws, and any other relatives.)
   - ☐1 Nine or more
   - ☐2 Five to eight
   - ☐3 Three or four
   - ☐4 Two
   - ☐5 One
   - ☐6 None

3. With how many relatives or family members can you comfortably discuss private matters? (Note: Include spouse, in-laws, and any other relatives.)
   - ☐1 Nine or more
   - ☐2 Five to eight
   - ☐3 Three or four
   - ☐4 Two
   - ☐5 One
   - ☐6 None

not for use without written copyright permission

The following three questions are about contacts with friends or neighbours.

4. How many friends/neighbours do you see or hear from <u>at least once a month</u>?
   - ☐1 Nine or more
   - ☐2 Five to eight
   - ☐3 Three or four
   - ☐4 Two
   - ☐5 One
   - ☐6 None

5. How many friends/neighbours do you feel close to that you can call on for help?
   - ☐1 Nine or more
   - ☐2 Five to eight
   - ☐3 Three or four
   - ☐4 Two
   - ☐5 One
   - ☐6 None

6. With how many friends/neighbours can you comfortably discuss private matters?
   - ☐1 Nine or more
   - ☐2 Five to eight
   - ☐3 Three or four
   - ☐4 Two
   - ☐5 One
   - ☐6 None

© 2000 This questionnaire is protected by copyright.

7. How often do you have someone who shows you love and affection if you need it?

- ☐1 Always
- ☐2 Very Often
- ☐3 Often
- ☐4 Sometimes
- ☐5 Seldom
- ☐6 Never

8. How often do you have someone to share your most private worries and fears with if you need it?

- ☐1 Always
- ☐2 Very Often
- ☐3 Often
- ☐4 Sometimes
- ☐5 Seldom
- ☐6 Never

9. How often do you have someone to love and make you feel wanted?

- ☐1 Always
- ☐2 Very Often
- ☐3 Often
- ☐4 Sometimes
- ☐5 Seldom
- ☐6 Never

10. How many times per month do you participate in social groups or organisations? *For example, hobby or recreational groups; community organisations including political or charity groups; and church or religious organisations.*

- ☐1 Nine or more
- ☐2 Five to eight
- ☐3 Three or four
- ☐4 Two
- ☐5 One
- ☐6 Zero

not for use without written copyright permission

Your General Health

1. All in all, would you say that your health is generally …

- ☐1 … Excellent
- ☐2 … Good
- ☐3 … Fair
- ☐4 … Poor

Functioning

Please indicate if you are <u>able</u> to do each of the following activities. If you do not do them, answer as if you tried to do them yourself.		Yes	Yes, with difficulty	Yes, with a device	Yes, with help from someone	No
1.	Feed yourself?	☐1	☐2	☐3	☐4	☐5
2.	Move from your bed to a chair or to standing up?	☐1	☐2	☐3	☐4	☐5
3.	Get yourself to the toilet?	☐1	☐2	☐3	☐4	☐5
4.	Dress yourself?	☐1	☐2	☐3	☐4	☐5
5.	Bathe yourself?	☐1	☐2	☐3	☐4	☐5
6.	Use the telephone?	☐1	☐2	☐3	☐4	☐5
7.	Get beyond walking distance of your home; for example, by driving or using public transport?	☐1	☐2	☐3	☐4	☐5
8.	Shop for groceries?	☐1	☐2	☐3	☐[illegible]	☐5
9.	Prepare meals?	☐[illegible]	☐[illegible]	☐3	☐4	☐5
10.	Do housework?	☐1	☐2	☐3	☐4	☐5
11.	Do D.I.Y. work?	☐1	☐2	☐3	☐4	☐5
12.	Do laundry?	☐1	☐2	☐3	☐4	☐5
13.	Take medicines?	☐1	☐2	☐3	☐4	☐5
14.	Manage money?	☐1	☐2	☐3	☐4	☐5
15.	Walk a quarter of a mile?	☐1	☐2	☐3	☐4	☐5
16.	Walk 1 mile at least 3 times a week?	☐1	☐2	☐3	☐4	☐5
17.	Participate in active sports enough to work up a sweat or become winded at least 3 times a week?	☐1	☐2	☐3		☐5

not for use without written copyright permission

© 2000 This questionnaire is protected by copyright.

For each activity listed above, you were asked if you were ***able*** *to do them. For each activity that follows, you are being asked whether you have regularly or permanently* ***changed the way*** *(questions 18-20) you do them or* ***decreased how often*** *(questions 21-23) you do them for health or physical reasons.*

**For health or physical reasons:**<br>**<u>In the past 12 months, have you changed the way you …</u>**		**No**	**Yes**	**Have not done in the past year**
18.	... **Walk ½ mile?**<br>*For example, you walk more slowly or carefully; you use a different stance or gait; you use a walkingstick, frame, or other aid; you take more frequent rest stops.*	☐1	☐2	☐3
19.	... **Climb 10 steps?**<br>*For example, you use the handrail more often; you reduce the number of steps taken at a time; you walk more slowly or carefully; you make more frequent rest stops.*	☐1	☐2	☐3
20.	... **Get into or out of a car or bus?**<br>*For example, you use the door or seat to help steady you; you rely more on your arms to help you; you enter or exit more slowly; you require help from others.*	☐1	☐2	☐3

not for use without written copyright permission

**For health or physical reasons:**<br>**<u>In the past 12 months, have you decreased how often you …</u>**		**No**	**Yes**	**Have not done in the past year**
21.	... **Walk ½ mile?**<br>*For example, you cut down from walking every day to walking 3-4 times per week because you tire more easily.*	☐1	☐2	☐3
22.	... **Climb 10 steps?**<br>*For example, you take the elevator more often whenever possible because of pain in your joints.*	☐1	☐2	☐3
23.	... **Get into or out of a car or bus?**<br>*For example, you decrease the number of car trips you take because it is difficult to get yourself out of the seat.*	☐1	☐2	☐3

© 2000 This questionnaire is protected by copyright.

Tobacco Use

This section is for ALL respondents.

1. Which best describes your cigarette smoking habits?
   - ☐ 1 I am presently a cigarette smoker
   - ☐ 2 I used to smoke cigarettes
   - ☐ 3 I never smoked cigarettes

2. Do you presently use tobacco in other forms (cigar or pipe)?
   - ☐ 1 No
   - ☐ 2 Yes

If you are a former cigarette smoker, ***skip to page 21 (question 8).***
If you never smoked cigarettes, ***skip to page 21 (Alcohol Use).***

This section is for CURRENT cigarette smokers only.

3. How many cigarettes do you <u>usually</u> smoke in a day?
   - ☐ 1 1 per day or less
   - ☐ 2 More than 1 per day ☐☐☐ cigarettes per day

4. How old were you when you <u>first</u> started smoking cigarettes daily (regularly)?
   ☐☐ years old

5. Have you seriously thought about quitting smoking in the next 6 months?
   - ☐ 1 No → *skip to question 7 on this page*
   - ☐ 2 Yes

6. Are you planning to quit smoking in the next 30 days?
   - ☐ 1 No
   - ☐ 2 Yes

7. Which of the following keep you from quitting smoking?
   Please tick all that apply and then skip to page 21 (Alcohol Use)
   - a. ☐ 1 I don't want to quit
   - b. ☐ 1 I don't think I can do it
   - c. ☐ 1 Stress
   - d. ☐ 1 Weight gain
   - e. ☐ 1 Other

not for use without written copyright permission

© 2000 This questionnaire is protected by copyright.

For FORMER cigarette smokers only:

8. How old were you when you first started smoking cigarettes (fairly regularly)?

☐☐ Years old

9. About how old were you when you last smoked cigarettes (fairly regularly)?

☐☐ Years old

Alcohol Use

Please count one drink of alcohol as equal to one of the following:

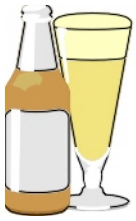

One half-pint or can of beer

or

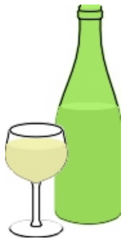

One normal wine glass or sherry glass

or

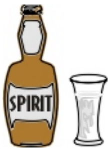

One pub measure of spirits

or

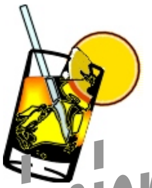

One cocktail containing a single measure of spirits

not for use without written copyright permission

1. How often do you have a drink containing alcohol?
   - ☐1 Never ➔ *Skip to page 23 (Physical Activity)*
   - ☐2 Monthly or less
   - ☐3 Two to four times a month
   - ☐4 Two to three times a week
   - ☐5 Four or more times a week

Please answer the following questions 2 to 10 even if you have a drink rarely

2. How many drinks containing alcohol do you have on a typical occasion when you drink?
   - ☐1 1 or 2
   - ☐2 3 or 4
   - ☐3 5 or 6
   - ☐4 7 to 9
   - ☐5 10 or more

3. How often do you have **four** or more drinks on one occasion?
   - ☐1 Never
   - ☐2 Less than monthly
   - ☐3 Monthly
   - ☐4 Weekly
   - ☐5 Daily or almost daily

© 2000 This questionnaire is protected by copyright.

4. During the last year, how often have you found that you were not able to stop drinking once you had started?

- ☐ 1 Never
- ☐ 2 Less than monthly
- ☐ 3 Monthly
- ☐ 4 Weekly
- ☐ 5 Daily or almost daily

5. During the last year, how often have you failed to do what was normally expected from you because of drinking?

- ☐ 1 Never
- ☐ 2 Less than monthly
- ☐ 3 Monthly
- ☐ 4 Weekly
- ☐ 5 Daily or almost daily

6. During the last year, how often have you needed a first drink in the morning to get yourself going after a heavy drinking session?

- ☐ 1 Never
- ☐ 2 Less than monthly
- ☐ 3 Monthly
- ☐ 4 Weekly
- ☐ 5 Daily or almost daily

7. During the last year, how often have you had a feeling of guilt or remorse after drinking?

- ☐ 1 Never
- ☐ 2 Less than monthly
- ☐ 3 Monthly
- ☐ 4 Weekly
- ☐ 5 Daily or almost daily

not for use without written copyright permission

8. During the last year, how often have you been unable to remember what happened the night before because you had been drinking?

- ☐ 1 Never
- ☐ 2 Less than monthly
- ☐ 3 Monthly
- ☐ 4 Weekly
- ☐ 5 Daily or almost daily

9. Have you been injured as a result of your drinking?

- ☐ 1 No
- ☐ 2 Yes, but not in the last year
- ☐ 3 Yes, during the last year

10 Has a relative, friend, doctor, or other health worker been concerned about your drinking or suggested that you should cut down?

- ☐ 1 No
- ☐ 2 Yes, but not in the last year
- ☐ 3 Yes, during the last year

© 2000 This questionnaire is protected by copyright.

Physical Activity

The following questions ask about your physical activities over the past 7 days. If the past 7 days were not typical for you, please answer for a typical week. Answer all items as accurately as possible. Please remember to complete both *parts of each question, if requested.*

1a. Over the past 7 days, how often did you participate in **sitting activities** *such as reading, watching TV or doing handicrafts*?

- ☐1 Never → *Skip to question 2a on this page*
- ☐2 1-2 days per week
- ☐3 3-4 days per week
- ☐4 5-7 days per week

1b. On average, how many hours per day did you engage in these sitting activities?

- ☐1 Less than 1 hour per day
- ☐2 1 but less than 2 hours per day
- ☐3 2 - 4 hours per day
- ☐4 More than 4 hours per day

2a. Over the past 7 days, how often did you take a **walk** outside your home or garden for any reason? *For example, for fun or exercise, walking to work, walking the dog, etc.?*

- ☐1 Never → *Skip to question 3a on this page*
- ☐2 1-2 days per week
- ☐3 3-4 days per week
- ☐4 5-7 days per week

2b. On average, how many hours per day did you spend walking?

- ☐1 Less than 1 hour per day
- ☐2 1 but less than 2 hours per day
- ☐3 2 - 4 hours per day
- ☐4 More than 4 hours per day

not for use without written copyright permission

3a. Over the past 7 days, how often did you engage in **light sport or recreational activities** *such as bowls, fishing, yoga, tai chi or other similar activities*?

- ☐1 Never → *Skip to page 24 (question 4a)*
- ☐2 1-2 days per week
- ☐3 3-4 days per week
- ☐4 5-7 days per week

3b. On average, how many hours per day did you engage in these light sport or recreational activities?

- ☐1 Less than 1 hour per day
- ☐2 1 but less than 2 hours per day
- ☐3 2 - 4 hours per day
- ☐4 More than 4 hours per day

© 2000 This questionnaire is protected by copyright.
© 1991 New England Research Institutes, Inc, 9, Galen Street, Watertown, MA 02472 U.S.A.

4a. Over the past 7 days, how often did you engage in **moderate sport or recreational activities** *such as ice skating, badminton, brisk walking, 10pin bowling, keep fit class or other similar activities*?

- ☐1 Never → *Skip to question 5a on this page*
- ☐2 1-2 days per week
- ☐3 3-4 days per week
- ☐4 5-7 days per week

4b. On average, how many hours per day did you engage in these moderate sport or recreational activities?

- ☐1 Less than 1 hour per day
- ☐2 1 but less than 2 hours per day
- ☐3 2 - 4 hours per day
- ☐4 More than 4 hours per day

5a. Over the past 7 days, how often did you engage in **strenuous sport or recreational activities** *such as jogging, swimming, cycling, tennis, aerobic class, skiing (downhill or cross-country) or other similar activities (including those using exercise machines)*?

- ☐1 Never → *Skip to question 6a on this page*
- ☐2 1-2 days per week
- ☐3 3-4 days per week
- ☐4 5-7 days per week

not for use without written copyright permission

5b. On average, how many hours per day did you engage in these strenuous sport or recreational activities?

- ☐1 Less than 1 hour per day
- ☐2 1 but less than 2 hours per day
- ☐3 2 - 4 hours per day
- ☐4 More than 4 hours per day

6a. *Another type of physical activity is muscle strengthening or conditioning.*
Over the past 7 days, how often did you do any **exercises** specifically to increase muscle strength and endurance, *such as lifting weights, doing pushups, using exercise machines for different muscle groups, etc.*?

- ☐1 Never → *Skip to page 25 (question 7)*
- ☐2 1-2 days per week
- ☐3 3-4 days per week
- ☐4 5-7 days per week

6b. On average, how many hours per day did you engage in exercises to increase muscle strength and endurance?

- ☐1 Less than 1 hour per day
- ☐2 1 but less than 2 hours per day
- ☐3 2 - 4 hours per day
- ☐4 More than 4 hours per day

© 2000 This questionnaire is protected by copyright.
© 1991 New England Research Institutes, Inc, 9, Galen Street, Watertown, MA 02472 U.S.A.

7.	**During the <u>past 7 days</u>, have you done any**	**No**	**Yes**
a.	Light housework, *such as dusting or washing dishes?*	☐1	☐2
b.	Heavy housework or chores, *such as vacuuming, scrubbing floors or washing windows, or carrying wood?*	☐1	☐2
c.	Home repairs *like painting, wallpapering, electrical work, etc.?*	☐1	☐2
d.	Heavy gardening?	☐1	☐2
e.	Light gardening?	☐1	☐2
f.	Caring for another person*, such as a child, dependent spouse, or another adult?*	☐1	☐2

8a. **<u>During the past 7 days</u>, did you work for pay or as a volunteer?**

☐1 No → *Skip to question 9 on this page*

☐2 Yes ↓

8b. **If *Yes***: How many <u>hours per week</u> did you work for pay and/or as a volunteer?

☐☐ Hours per week

not for use without written copyright permission

8c. **If *Yes***: Which of the following categories best describes **the amount of physical activity required** on your job and/or volunteer work?

☐1 Mainly sitting with slight arm movements *(for example: office worker, watchmaker, seated assembly line worker, bus driver, etc.).*

☐2 Sitting or standing with some walking *(for example: cashier, general office worker, light tool and machinery worker).*

☐3 Walking, with some handling of heavy weight *(for example: postman, waiter/waitress, construction worker, heavy tool and machinery worker)*.

☐4 Walking and heavy manual work often requiring handling of heavy materials *(for example: construction or building worker).*

9. Do you participate in some form of light, moderate or strenuous physical activity <u>on most days,</u> such as those described in the questions above?
Include both work and non-work related activity.

☐1 Yes, I have been for more than 6 months

☐2 Yes, I have been but for less than 6 months

☐3 No, but I plan to in the next month

☐4 No, but I plan to in the next 6 months

☐5 No, and I do not intend to in the next 6 months

© 2000 This questionnaire is protected by copyright.
© 1991 New England Research Institutes, Inc, 9, Galen Street, Watertown, MA 02472 U.S.A.

10. Which of the following keep you from participating more in physical activity?
Please tick ALL that apply.

a. ☐1 I already exercise frequently and regularly
b. ☐1 I don't have anyone to exercise with
c. ☐1 There is nowhere to exercise
d. ☐1 I don’t have time/don’t get around it
e. ☐1 Cost
f. ☐1 Weather
g. ☐1 I have an illness limiting my physical activity
h. ☐1 I have a physical limitation
i. ☐1 I have pain with physical activity
j. ☐1 Other reasons

Nutrition

Think about your eating habits <u>over the past month</u>. About how often do you eat food in each of the following categories?
In your answer, exclude non-fat versions of these foods
Tick ONE answer for each food category.

not for use without written copyright permission

	**How often do you eat . . .?**	**Never or less than once per MONTHS**	**[illegible]-3 times per MONTHS**	**1-2 times per WEEK**	**3-[illegible] times per WEEK**	**5 times or more per WEEK**
1.	Hamburgers, cheeseburgers, minced beef	☐1	☐2	☐3	☐4	☐5
2.	Beef or pork, such as steak, roasts, chops	☐1	☐2	☐3	☐4	☐5
3.	Fried chicken with skin, fried fish	☐1	☐2	☐3	☐4	☐5
4.	Hot dogs, frankfurters, or other sausages	☐1	☐2	☐3	☐4	☐5
5.	Cold cuts, lunch meats (salami, etc.)	☐1	☐2	☐3	☐4	☐5
6.	Salad cream (not diet) or mayonnaise	☐1	☐2	☐3	☐4	☐5
7.	Margarine, butter or gravy	☐1	☐2	☐3	☐4	☐5
8.	Eggs	☐1	☐2	☐3	☐4	☐5
9.	Bacon, ham	☐1	☐2	☐3	☐4	☐5
10.	Cheese spread or cheese (not low-fat)	☐1	☐2	☐3	☐4	☐5

© 2000 This questionnaire is protected by copyright.

How often do you eat . . .?		Never or less than once per MONTHS	1-3 times per MONTHS	1-2 times per WEEK	3-4 times per WEEK	5 times or more per WEEK
11.	Whole milk, semi-skimmed milk or cream	☐1	☐2	☐3	☐4	☐5
12.	French fries, fried potatoes, or chips	☐1	☐2	☐3	☐4	☐5
13.	Crisps, popcorn, cracker biscuits	☐1	☐2	☐3	☐4	☐5
14.	Ice cream (not low-fat)	☐1	☐2	☐3	☐4	☐5
15.	Doughnuts, pastries, pies, cake, cookies, biscuits	☐1	☐2	☐3	☐4	☐5

How often do you eat . . .?		Never or less than once per WEEK	About 1time per WEEK	2-3 time per WEEK	4-6 time per WEEK	Every day
16.	Orange juice or other 100% fruit juices	☐1	☐2	☐3	☐4	☐5
17.	Fruit (do not count juice)	☐1	☐2	☐3	☐4	☐5
18.	Green salad	☐1	☐2	☐3	☐4	☐5
19.	Potatoes	☐1	☐2	☐3	☐4	☐5
20.	Beans, baked beans, kidney beans	☐1	☐2	☐3	☐4	☐5
21.	Other vegetables	☐1	☐2	☐3	☐4	☐5
22.	High-fibre or bran cereal	☐1	☐2	☐3	☐4	☐5
23.	Dark bread, such as whole wheat or rye	☐1	☐2	☐3	☐4	☐5
24.	Whole-grain rice or whole-grain pasta	☐1	☐2	☐3	☐4	☐5

not for use without written copyright permission

© 2000 This questionnaire is protected by copyright.

25. Do you consistently take steps to limit eating high-fat foods?

- ☐1 Yes, I have been for more than 6 months
- ☐2 Yes, I have been but for less than 6 months
- ☐3 No, but I plan to in the next month
- ☐4 No, but I plan to in the next 6 months
- ☐5 No, and I do not intend to in the next 6 months

26. Which of the following keep you from eating less fat?
Please tick ALL that apply.

a. ☐1 I already minimise fat intake
b. ☐1 I don't think it's important to eat less fat
c. ☐1 I like the taste of high-fat foods
d. ☐1 My friends/family members don't want to cut down on high-fat foods
e. ☐1 It is too much trouble to shop for or prepare low-fat foods
f. ☐1 High-fat foods are easily available
g. ☐1 Low-fat items are more expensive
h. ☐1 Other

27. Do you consistently take steps to include fruits, vegetables and fibre into your diet?

- ☐1 Yes, I have been for more than 6 months
- ☐2 Yes, I have been but for less than 6 months
- ☐3 No, but I plan to in the next month
- ☐4 No, but I plan to in the next 6 months
- ☐5 No, and I do not intend to in the next 6 months

not for use without written copyright permission

28. Which of the following keep you from increasing your fruit, vegetable and fibre intake?
Please tick ALL that apply

a. ☐1 I already eat plenty of fruits, vegetables and fibre
b. ☐1 I don't think it's important to add them to my diet
c. ☐1 I don't like the taste of fruits and vegetables
d. ☐1 My friends/family members don't want me to include more of them
e. ☐1 It's too much trouble to shop for or prepare fruits, vegetables and fibre
f. ☐1 Good produce is not easily available
g. ☐1 Good produce is expensive
h. ☐1 Other

© 2000 This questionnaire is protected by copyright.

Injury Prevention

Motor vehicle safety

1. How often do you forget to use safety belts when you drive or ride in a car?
   - ☐1 Never forget
   - ☐2 Seldom forget
   - ☐3 Sometimes forget
   - ☐4 Nearly always forget
   - ☐5 Always forget

2. How often did you worry when you were a driver (or a passenger) in a car that you (or the driver) had too much alcohol to drink?
   - ☐1 Never
   - ☐2 Less than once a year
   - ☐3 Less than once a month, but at least once a year
   - ☐4 Less than once a week, but at least once a month
   - ☐5 One or two times a week
   - ☐6 Three or more times a week
   - ☐7 Daily

Balance

not for use without written copyright permission

3. Do you limit your activities because you are afraid you will fall?
   - ☐1 No
   - ☐2 Yes

4. Have you ever fallen and not been able to get up?
   - ☐1 No
   - ☐2 Yes

5a. <u>During the past 12 months</u> have you fallen to the ground or floor?
   - ☐1 No → *Skip to page 30 (Preventive Care)*
   - ☐2 Yes ↓

5b. How many times did you fall
   - ☐1 One time
   - ☐2 More than one time

5c. Was the fall (were the falls) indoors or out of doors?
   - ☐1 Indoors
   - ☐2 Outdoors
   - ☐3 Indoors and outdoors

© 2000 This questionnaire is protected by copyright.

Preventive Care

Have you had a ...		No	Yes
1.	Blood pressure measurement within the past year?	☐1	☐2
2.	Eyesight checkup within the past year?	☐1	☐2
3.	Hearing checkup within the past year?	☐1	☐2
4.	Dental checkup within the past year?	☐1	☐2
5.	Cholesterol measurement within the past 5 years?	☐1	☐2
6.	Fasting blood glucose measurement within the past 3 years?	☐1	☐2
7.	Colon cancer screen within the past year?	☐1	☐2
8.	Immunisation against influenza ("flu jab") within the past year?	☐1	☐2
9.	Immunisation against pneumonia ("Pneumovax") within the past 10 years?	☐1	☐2
For Women Only:			
10.	Mammogram within the [illegible] years?	☐1	☐2
11.	Cervical smear within the past [illegible] years?	☐1	☐2

not for use without written copyright permission

12. For those preventive services listed above that you have not had, which of the following keep you from having them?
Please tick ALL that apply
a. ☐1 I have already had these preventive services
b. ☐1 I have no need to; I have no health problems
c. ☐1 I've never thought about it
d. ☐1 Financial reasons/cost, insurance
e. ☐1 I don't think it's important
f. ☐1 My general practitioner never recommended it
g. ☐1 I don't have the time/don't get around to it
h. ☐1 Other

© 2000 This questionnaire is protected by copyright.

Occupation & Retirement

1. What type of work did you do <u>most</u> of your life?
   Please tick only ONE response.
   - ☐ 1 Managerial and Professional
   - ☐ 2 Technical, Sales, and Administrative Support Occupations
   - ☐ 3 Service Occupations
   - ☐ 4 Farming, Forestry, and Fishing Occupations
   - ☐ 5 Precision Production, Craft, and Repair Occupations
   - ☐ 6 Machine Operators, Factory or Labouring
   - ☐ 7 Military Occupations
   - ☐ 8 Housewife
   - ☐ 9 Volunteer Work
   - ☐ 10 Other

2. Are you <u>currently</u> retired?
   - ☐ 1 No → *Skip to question 4 on this page*
   - ☐ 2 Yes ↓

3. Did you retire mainly because you thought your work would cause a health problem?
   - ☐ 1 No
   - ☐ 2 Yes

4. Are you <u>currently</u> working for pay?
   - ☐ 1 No
   - ☐ 2 Yes, part time
   - ☐ 3 Yes, full time

5. Are you <u>currently</u> doing unpaid work?
   - ☐ 1 No
   - ☐ 2 Yes, part time
   - ☐ 3 Yes, full time

not for use without written copyright permission

© 2000 This questionnaire is protected by copyright.

About You

1. Where were you born?
   - ☐1 Britain: Which place and which county?

     place ______________________ county ______

   - ☐2 In some other country: Which country?

     ____

2. What is your racial/ethnic background?
   - ☐1 White UK
   - ☐2 White Irish (non UK)
   - ☐3 Black Caribbean
   - ☐4 Black African
   - ☐5 Black other
   - ☐6 Indian
   - ☐7 Pakistani
   - ☐8 Bangladeshi
   - ☐9 Chinese
   - ☐[illegible] Other (please specify) ______________

3. What are your current living arrangements?
   - ☐1 I live alone
   - ☐2 I live with my spouse
   - ☐3 I live with someone else

4. Please describe your living location? I live in a …
   - ☐1 Private flat/home
   - ☐2 Residential Care or Residential Home
   - ☐3 Nursing Home

5. At what age did you complete your education in school or college?
   - ☐1 Before the age of 15 years
   - ☐2 At the age of 15 or 16 years
   - ☐3 Between the age of 17 and 20 years
   - ☐4 After the age of 21 years

6. Do you receive only the state pension?
   - ☐1 Yes
   - ☐2 No

not for use without written copyright permission

© 2000 This questionnaire is protected by copyright.

Questionnaire Evaluation Form

This brief survey asks questions about your recent experience completing the Older Persons Health Profile Questionnaire. Your honest responses will help us improve it for future use with others.

1. In general, how difficult was it for you to <u>understand</u> the questions and instructions in the Questionnaire?
   - ☐ 1 Very difficult
   - ☐ 2 Somewhat difficult
   - ☐ 3 Somewhat easy
   - ☐ 4 Very easy

If you answered **Very difficult** or **Somewhat difficult**, what sections, questions, or instructions did you find difficult to understand?

___

2. In general, how difficult was it for you to <u>complete</u> the Questionnaire?
   - ☐ 1 Very difficult
   - ☐ 2 Somewhat difficult
   - ☐ 3 Somewhat easy
   - ☐ 4 Very easy

not for use without written copyright permission

If you answered **Very difficult** or **Somewhat difficult**, what sections or questions did you find difficult to complete?

___

3. Please mark the response that best corresponds to the assistance you received in completing this questionnaire.
   - ☐ 1 Someone else completed the Questionnaire for me.
   - ☐ 2 I completed it with assistance from someone else.
   - ☐ 3 I competed it without assistance, but would have liked assistance.
   - ☐ 4 I completed it without assistance, and not feel I needed assistance .

4. How would you describe the <u>length</u> of the Questionnaire?
   - ☐ 1 Too long
   - ☐ 2 About right
   - ☐ 3 Too short

5. About how much <u>time</u> did it take you to complete the Questionnaire?

   ☐☐☐ minutes

© 2000 This questionnaire is protected by copyright.

6. Did you dislike any specific sections or questions on the Questionnaire?
   - ☐1 No
   - ☐2 Yes

If you answered Yes, what sections or questions did you dislike?

---

7. Are there any topic areas that were not covered in the Questionnaire that you think should have been covered in the Questionnaire?
   - ☐1 No
   - ☐2 Yes

If you answered Yes, what topic areas should have been covered?

Please take a moment to look back and make sure you have not missed any pages or questions.

Date Completed: Day ☐☐ Month ☐☐ Year ☐☐☐☐

not for use without written copyright permission

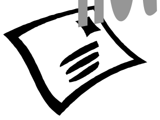

Would you kindly return this questionnaire within 20 days using the enclosed stamped addressed envelope.

Thank you for completing this survey!

Version 2.5: This questionnaire was developed by the Geriatric Research Unit Bern (Spital Bern Ziegler, Morillonstrasse 75, CH-3001 Bern/Switzerland) in collaboration with the Department of Health Care of the Elderly, King's College London, the Department of Primary Care and Population Sciences, University College London, and other organisations based on a license to use granted by the Regents of the University of California, Los Angeles. This questionnaire is protected by copyright and may not (as a whole or in part) be reproduced or used for other purposes without prior written permission of the copyright owners.
Version 2.5: © 2000, Regents of the University of California, Los Angeles.
Section "Physical activity": © 1991 New England Research Institutes, 9 Galen Street, Watertown, MA 02472 USA.
